# An integrated pan-cancer analysis of PSAT1: A potential biomarker for survival and immunotherapy

**DOI:** 10.3389/fgene.2022.975381

**Published:** 2022-08-29

**Authors:** Mingtao Feng, Huanhuan Cui, Wenjing Tu, Liangdong Li, Yang Gao, Lei Chen, Deheng Li, Xin Chen, Fengfeng Xu, Changshuai Zhou, Yiqun Cao

**Affiliations:** ^1^ Department of Neurosurgery, Fudan University Shanghai Cancer Center, Shanghai, China; ^2^ Department of Neurosurgery, Naval Medical Center of PLA, Shanghai, China

**Keywords:** PSAT1, bioinformatics analysis, immunotherapy, cancer, prognosis

## Abstract

Phosphoserine aminotransferase 1 (PSAT1) may be an oncogene that plays an important role in various cancer types. However, there are still many gaps in the expression of PSAT1 gene and its biological impact in different types of tumors. Here, we performed an integrated pan-cancer analysis to explore the potential molecular mechanisms of PSAT1 in cancers. We found that most human tumors express higher levels of PSAT1 than normal tissues, and that higher PSAT1 expression is associated with worse prognosis in Lung adenocarcinoma (LUAD), Pan-kidney cohort (KIPAN) and breast invasive carcinoma (BRCA), etc. In BRCA cases, the prognosis of patients with altered PSAT1 was worse than that of patients without alteration. In addition, PSAT1 hypermethylation is associated with T cell dysfunction and shortened survival time in BRCA. The Gene Set Enrichment Analysis (GSEA) analysis showed that PSAT1 can be enriched into the classic signaling pathways of cancer such as mTORC1 signaling, MYC targets and JAK STAT3. Further analysis demonstrated that PSAT1 was enriched in immune related signaling pathways in LUAD and BRCA. The results of immunoassay showed that PSAT1 was associated with immune cell infiltration in multiple cancer species. Furthermore, expression of PSAT1 was correlated with both tumor mutational burden (TMB) and microsatellite instability (MSI) in BRCA. Additionally, a remarkable correlation was found between PSAT1 expression and TMB in LUAD, and the expression of PSAT1 was negatively correlated with the Tumor Immune Dysfunction and Exclusion (TIDE) value, suggesting a good effect of immunotherapy. Together, these data suggest that PSAT1 expression is associated with the clinical prognosis, DNA methylation, gene mutations, and immune cell infiltration, contributing to clarify the role of PSAT1 in tumorigenesis from a variety of perspectives. What’s more, PSAT1 may be a new biomarker for survival and predicting the efficacy of immunotherapy for LUAD and BRCA.

## 1 Introduction

The development, invasion and metastasis of malignant tumors have very complex mechanisms. With the deepening of research, people have a certain understanding of the pathogenesis of some tumors, and the immunotherapy of malignant tumors has made a certain breakthrough. However, at present, there are still many gaps in the understanding of malignant tumors. The poor efficacy of immunotherapy for some tumors has brought great challenges to clinical treatment (Remon et al., 2020). This is mainly due to the lack of specific markers for diagnosis, prognosis and immunotherapy efficacy prediction. Therefore, it is of great clinical and practical significance to analyze the pan cancer expression of genes of interest and evaluate their relationship with clinical prognosis, potential molecular mechanisms and immune efficacy prediction.

PSAT1 belongs to the family of class V aminotransferases. It is an important rate limiting enzyme in the serine-glycine synthesis pathway, and glycine is an significant nutrient for the proliferation of malignant tumor cells. To convert 3-phosphohydroxypyruvate into L-phosphoserine, PSAT1 participates in the glutamate-linked transamination reaction, which is the second step of the serine-glycine biosynthetic pathway (Basurko et al., 1999). Song Gao et al. found that PSAT1 is significantly up-regulated in ER negative breast cancer and this up-regulation was able to enhance the proliferation of ER-negative breast cancer cells *in vitro* via the GSK3β/β-catenin/cyclin D1 pathway (Gao et al., 2017). In addition, Jun Dai et al. found that overexpression of microRNA-195–5p reduces cisplatin resistance and angiogenesis in ovarian cancer by inhibiting the PSAT1-dependent GSK3β/β-catenin signaling pathway (Dai et al., 2019). More and more evidence showed that PSAT1 may be a biomarker of many cancers as an oncogene (Chan et al., 2020; Metcalf et al., 2020; Wang et al., 2020). However, beyond the limited information provided by these studies, the role of PSAT1 in malignant tumors is still unclear.

In this study, we used the data available in the public database to conduct a comprehensive pan cancer analysis of PSAT1 to determine the correlation between its expression and the prognosis of various malignant tumors. In addition, we also performed bioinformatics analysis to determine the relationship between the expression of PSAT1 and promoter methylation, gene mutation, as well as tumor immune microenvironment and immunotherapy efficacy prediction in a variety of human tumors.

## 2 Materials and methods

**FIGURE 1:**
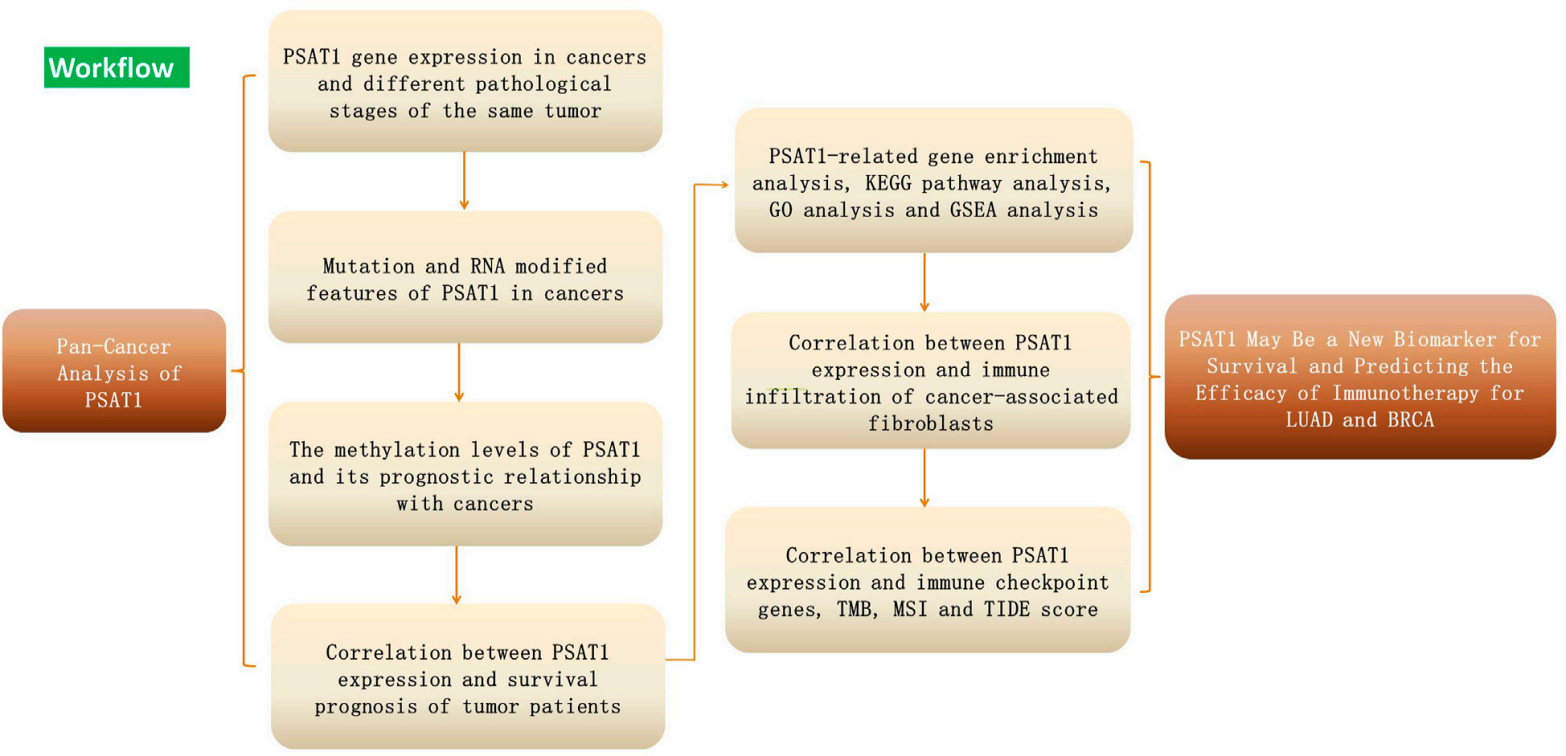
Workflow plot of the study design.

Figure 1 showed the workflow of this study.

### 2.1 Description of the PSAT1

The variant information of PSAT1 was derived from the PROTTER software tool (Omasits et al., 2014). (https://wlab.ethz.ch). A collection of Immunostaining images of PSAT1 protein localization was obtained from the Human Protein Atlas (https://www.proteinatlas.org) (Uhlén et al., 2015), We used the immunofluorescence staining images of two human cancer cell lines (A-431, U251 and U-2OS) to show the subcellular localization of PSAT1 in cancer cells. Then we collected expression profiles under physiological conditions of PSAT1 from GeneCards (http://www.genecards.org). By integrating genetic, omics, and chemical data, the OPENTARGET platform (https://www.targetvalidation.org) assists in identifying gene roles in disease and aids in the systematic identification of drug targets and prioritization (Carvalho-Silva et al., 2019).

### 2.2 Data processing and differential expression analysis

RNA sequencing and modification, somatic mutation, epigenetic and transcriptomic data and related clinical data were downloaded from normalized data sets: The Cancer Genome Atlas (TCGA) TARGET GTEx (PANCAN, *n* = 19,131, *g* = 60,499) using UCSC Xena (https://xena.ucsc.edu/), from which we further extracted the ENSG00000135069 gene (PSAT1). The expression of PSAT1 was evaluated in 34 normal tissues and matched standard samples in 34 cancers using the downloaded data and expression levels compared between cancer samples. Through the module of “Pathological staging map” of GEPIA2, the violin map of PSAT1 expression of TCGA tumors we obtained in different pathological stages into box violin map expression data. To assess the difference in PSAT1 expression at the protein level. A comparison of PSAT1 expression in primary tumors and normal tissues was conducted with UALCAN (http://ualcan.path.uab.edu/index.htm). From the Human Protein Atlas (HPA), images of the expression of PSAT1 protein in seven tumor tissues and the corresponding normal tissues have been downloaded and analyzed. Expression data were Log2 [TPM (PSAT1) +1] transformed and two sets of t-tests conducted on these tumor types; *p* < 0.05 were considered to indicate differential expression between tumor and normal tissues. Data analysis was conducted using R software (Version 4.0.2; https://www.Rproject.org).

### 2.3 Genetic alteration analysis

With a web browser, we went to the cBioPortal website (https://www.cbioportal.org/), selected the TCGA Pan Cancer Atlas Studies section, and used PSAT1 to search for genetic alterations (Cerami et al., 2012; Gao et al., 2013). The “Cancer Type Summary” module provides results for mutations and copy number variations (CNAs) of the PSAT1 gene. With the Mutation module, information on the mutation sites of PSAT1 can be displayed in a protein schematic or in a three-dimensional (3D) structure. In addition, in BRCA cases with and without PSAT1 mutation, we determined differences in overall survival (OS), disease-free survival (DFS) and progression-free survival (PFS) by using the “Compare” module. Meanwhile, the log-rank *p*-value Kaplan-Meier graph is generated.

### 2.4 RNA modification correlated analysis

We divided all related genes according to RNA modification types (N1-methyladenosine, 5-methylcytosine and N6-methyladenosine modifications) and the types of regulators (writer, reader and eraser) into two groups (Nombela et al., 2021). The correlation between gene expression of PSAT1 and related genes in cancer was analyzed using Spearman’s method. Different colors represent correlation coefficients in heat maps, which are divided into horizontal and vertical axes based on cancer types and gene markers.

### 2.5 Epigenetic methylation analysis

As part of UALCAN’s interactive web resource, using the TCGA methylation module, we compared methylation levels among normal tissues and tumor tissues. By measuring its beta value, a promoter is assessed for methylation status, which ranges from zero (unmethylated) to one (fully methylated). Different β cut-off points indicate hypermethylation (b: 0.7–0.5) and hypomethylation (b: 0.3–0.25), respectively (Shinawi et al., 2013; Men et al., 2017). Moreover, we used TIDE server to analyze the effects of methylation on the phenotype and prognosis of dysfunctional T cells.

### 2.6 Survival prognosis analysis

Related prognostic information, including OS time and PFS time were also downloaded from the UCSC Xena database except breast and lung cancers, which were generated using the Kaplan–Meier Plotter website. The cohorts were split according to expression levels using a 50% cutoff value for high and 50% for low. Finally, the log-rank *p* value of the K-M method and hazard ratio (HR) with a 95% confidence interval (95% CI) were computed, and the outcomes were summarized and presented in Supplementary Tables. The significant association of PSAT1 expression with worse or better prognosis was further validated in survival curves.

### 2.7 PSAT1-related gene enrichment analysis

The PPI network was created using the STRING (https://string-db.org/) website to screen 50 experimentally verified proteins binding to PSAT1. PSAT1 and selected genes were analyzed by the Correlation Analysis Module of GEPIA2. The point plot uses LOG2 TPM. *p* values and correlation coefficients (R) are indicated. For the correlation heatmap in Figure 5B, we used purity-adjusted Spearman rank correlation to provide partial correlation (cor) and *p* values for selected genes in the “Gene_Corr” module containing TIMER2. The mRNA expression levels and the subtypes of cancer are all from RNA-Seq on TCGA. Especially, TCGA BRCA subtypes, based on the PAM50 gene set including LumA, LumB, Her2, and Basal subtypes were from https://github.com. The combined gene lists are then uploaded to DAVID for analysis, using the Functional Annotation Tool. With the Cluster Profiler R package (v3.6.0), the Kyoto Encyclopedia of Genes and Genomes (KEGG) pathway enrichment analysis was conducted. GSEA was employed to investigate the potential biological process of PSAT1 in pan-cancer. GSEA was performed using R packages “cluster profiler” (Yu and He, 2016). Adjusted *p* values <0.05 were considered statistically significant. Gene-sets used in Figure 6E were c7 Immune Sig DB, Supplementary Figure S3 were Hallmarks v7.2 from GSEA Molecular Signatures Database.

### 2.8 Immune infiltration and cell type-level expression analysis

By using the TIMER2 server, we assessed the correlation of PSAT1 expression and immune cells, including CD4^+^ T cell clusters, CD8^+^ T cells, dendritic cells (DCs), macrophages, neutrophils, and B cells across all TCGA tumors. The data is visualized as heat map. *p* values and partial correlation coefficients (CORs) were derived by adjusting the Spearman rank correlation. Moreover, PSAT1 expression and tumor purity were investigated. To estimate the abundance of tumor infiltrating immune cells, a previously published statistical deconvolution methodology was applied [18]. Then, we selected testicular germ cell tumors (TGCT), lung squamous cell carcinoma (LUSC), BRCA, glioblastoma (GBM), low grade glioma (LGG) and bladder urothelial carcinoma (BLCA), which are highly correlated with immune cells, to make scatter plots. According to the correlation between immune cell types and cancer species, the expression of immune cell subtypes in the different types of cancer was also examined. The PSAT1 expression and cell type-level expression was analyzed using GEPIA1 (GEPIA 2021. cancer-pku. cn).

### 2.9 Immune modulator genes correlation analysis

We extracted from each tumor sample the expression data of 60 marker genes of two types of immune modulator genes (24 Inhibitory and 36 Stimulatory), and further we screened the samples for the following sources: Primary Solid Tumor, Primary Tumor, Primary Blood Derived Cancer-Bone Marrow, Primary Blood Derived Cancer-Peripheral Blood. We also filtered all normal samples, and furthermore, for each next, we generated a pearson correlation analysis heatmap of the marker genes of the two types of immune modulator genes and PSAT1 gene expression in various cancers. In the heat map, cancer types are displayed horizontally, immune genes are displayed vertically, and correlation coefficients are displayed as colors.

### 2.10 TMB, MSI and TIDE correlated analysis

In order to determine whether PSAT1 gene expression was related to TMB or MSI scores, we downloaded the simple nucleoside variation data set of level4 of all TCGA samples processed by mutect2 software (doi:10.1038/nature08822) from GDC（https://portal.gdc.cancer.gov/）. The samples with expression level of 0 and cancer species with less than 3 samples in a single cancer species were filtered. Log2 (x+0.001) transformation was carried out for each expression value. Finally, the expression data of PSAT1 of 37 cancer species were obtained. Next, the TMB function of R software package maftools (version 2.8.05) was used to calculate the TMB of each tumor. In addition, we obtained the MSI score of each tumor from the previous study (Bonneville et al., 2017). Eventually, we integrated and analyzed the expression of PSAT1, TMB and MSI. PSAT1, TMB and MSI are shown in the horizontal axis of the graph, and cancer types are shown in the vertical axis. There are different types of cancer represented by different colors of dots in the graph and the dots size represents the magnitude of the correlation coefficients, with diverse colors indicating *p*-values. To evaluate PSAT1 gene expression in tumor samples and the possibility of tumor immune escape, TIDE score was calculated online (http://tide.dfci.harvard.edu/). TIDE score was used to evaluate clinical efficacy in decision making of immune checkpoint inhibition therapy. TIDE score between groups was compared by the Student’s t test (Jiang et al., 2018).

## 3 Results

### 3.1 Physiological characteristics of PSAT1 variants, localization, and expression profiles

The PSAT1 protein topology revealed no obvious mutation (Supplementary Figure S1A). To study the intracellular localization of PSAT1, we used indirect immunofluorescence to evaluate the distribution of PSAT1 in the endoplasmic reticulum (ER), nucleus and microtubules of A-431 (Human epidermal carcinoma), U-251MG (Human astroglioma cells) and U-2 osteosarcoma cells. Our analysis results showed that PSAT1 overlapped with ER markers in above 3 cell lines, which indicated the subcellular localization of PSAT1 in ER. In other words, there is no expression of PSAT1 in the nucleus or microtubules (Supplementary Figure S1B). We also analyzed the distribution of PSAT1 mRNA in normal tissues, the results indicated that it was distributed in immune, nervous system, secretory, muscle and reproductive tissues (Supplementary Figure S1C). In addition, by analyzing the gene and disease network interaction, we found its related diseases, including neurological diseases, nutritional or metabolic diseases, and congenital diseases (Supplementary Figure S1D).

### 3.2 Gene expression analysis data

**FIGURE 2:**
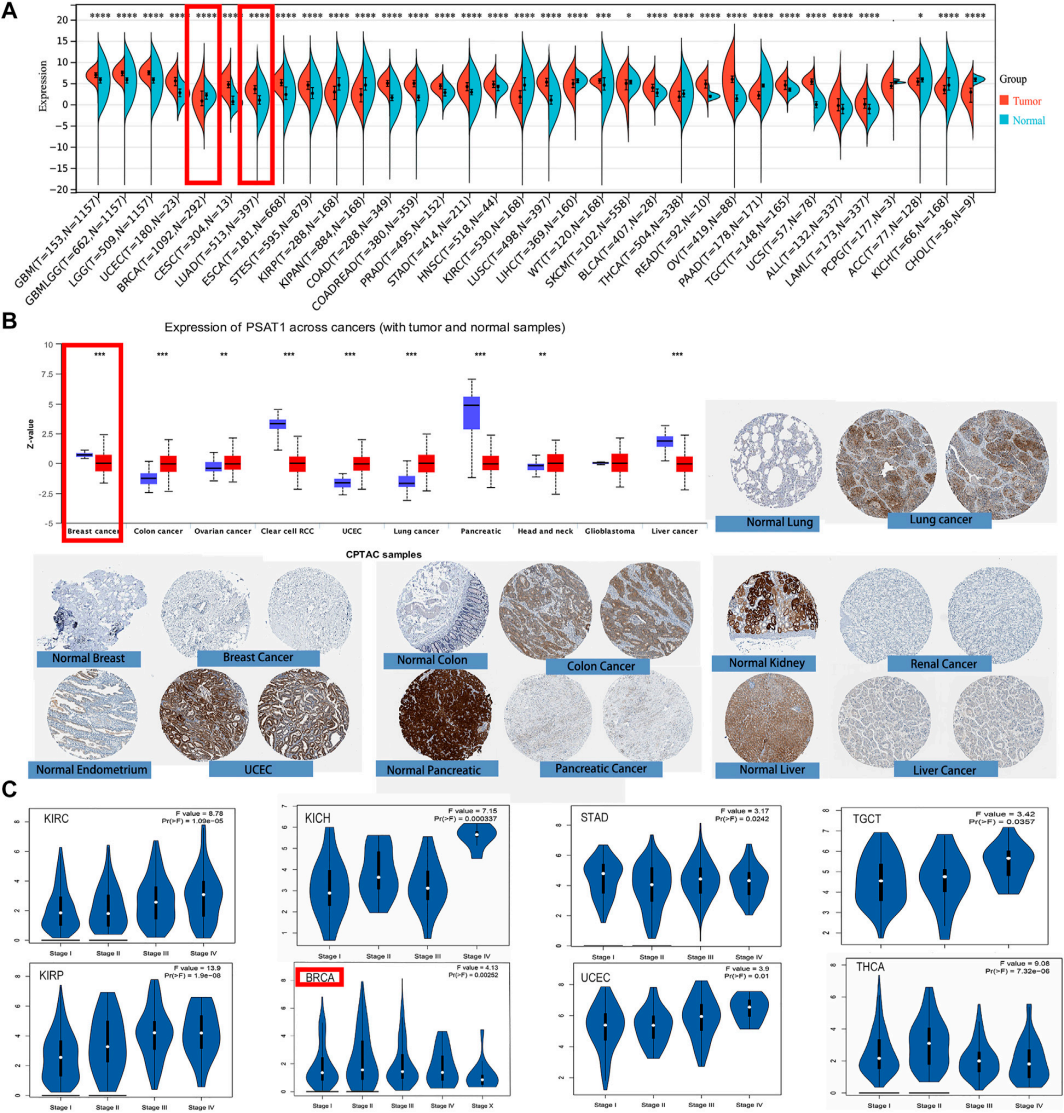
PSAT1 gene expression in cancers and different pathological stages of the same tumor. **(A)**Increased or decreased expression of PSAT1 in different cancers or specific cancer subtypes through TIMER2. *p < 0.05; ***p < 0.001; ***p < 0.0001. **(B)** PSAT1 total protein expression between normal and primary tissues of breast cancer, colon cancer, ovarian cancer, clear cell RCC, UCEC, lung cancer, pancreatic cancer, head and neck cancer, GBM and liver cancer through the CPTAC dataset. **p < 0.01; ***p < 0.001. **(C)** Analyzing the expression level differences of PSAT1 in different pathological stages of BRCA, KICH, KIRC, KIRP, STAD, TGCT, THCA, UCEC through TCGA database.

First, we used TIMER2 to compare the mRNA expression profiles and the expression level of PSAT1 in each type of tumor and their corresponding normal tissue. According to Figure 2A, the expression level of PSAT1 in the tumor tissues of BLCA, Cervical squamous cell carcinoma and Endocervical adenocarcinoma (CESC), Colon adenocarcinoma (COAD), Esophageal carcinoma (ESCA), Head and neck squamous cell carcinoma (HNSC), LUAD, LUSC, Prostate adenocarcinoma (PRAD), Rectum adenocarcinoma (READ), Stomach adenocarcinoma (STAD), and Uterine Corpus Endometrial Carcinoma (UCEC) is higher than the corresponding normal control tissues. However, the expression of PSAT1 is lower in Acute Myeloid Leukemia (LAML), Cholangiocarcinoma (CHOL), Kidney renal clear cell carcinoma (KIRC), Kidney renal papillary cell carcinoma (KIRP) than the corresponding normal tissues. Generally, most human tumors expressed PSAT1 at a higher level than normal.

By analyzing the CPTAC data set, we were also able to analyze the expression of PSAT1 total protein. Primary tissues from cancerous tumors such as ovarian cancer, colon cancer, UCEC, lung cancer, and head and neck cancer had higher expression levels than normal tissues. On the other hand, we also discovered that primary tissues of breast cancer, liver cancer, clear cell renal cancer, and pancreatic cancer have lower levels of PSAT1 total protein expression (Figure 2B).

Additionally, we examined differences in the expression of PSAT1 within the same cancer at different stages after analyzing PSAT1 expression in different cancer types. For this reason, the “Pathological Stage Plot” module of GEPIA2 was employed to investigate the correlation of PSAT1 expression and cancer pathological stages. PSAT1 expression differs significantly in BRCA, KIRC, Kidney Chromophobe (KICH), KIRP, STAD, TGCT, thyroid carcinoma (THCA) and UCEC in different stages, but it is particularly pronounced in KIRP (Figure 2C). This suggests that we need to dynamically observe the expression of PSAT1 in different stages of the same tumor and PSAT1 may have different roles in the progression of the tumor.

### 3.3 The genetic alteration and RNA modification of PSAT1 in pan-cancer datasets

**FIGURE 3:**
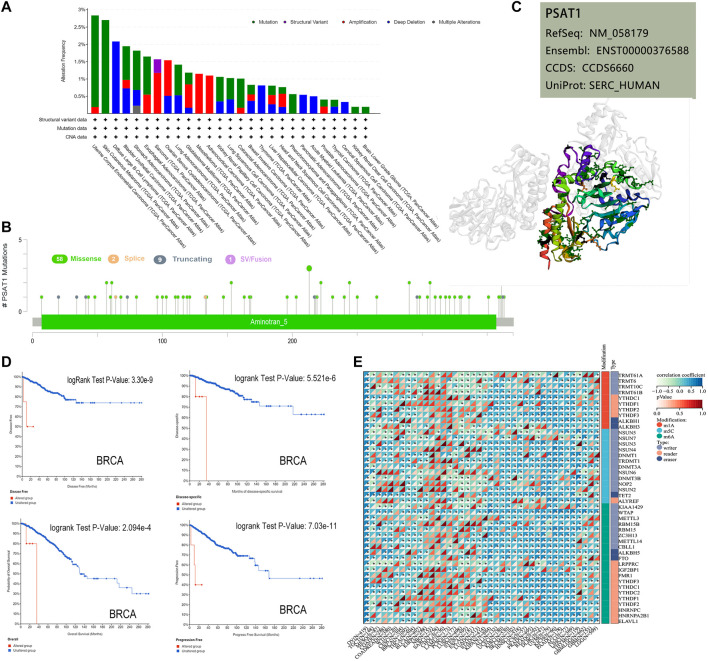
Mutation and RNA modified features of PSAT1 in cancers of TCGA. **(A)** Bar plot of PSAT1 alteration frequency with mutation type and **(B)** mutation site through cBioPortal tool. **(C)** The highest alteration frequency (R213HC) in the 3D structure of PSAT1 was displayed. **(D)** Analyzing the relationship between different mutation states and overall, disease-specific, disease-free and progression-free survival using the cBioPortal tool. **(E)** Correlation analyses of PSAT1 and three kinds of RNA modified (m1A (Carvalho-Silva et al., 2019), m5C (Nombela et al., 2021), m6A (Vié et al., 2008)) marker genes in Pan-Cancer. *p < 0.05.

Based on TCGA database, we examined the status of genetic alteration of PSAT1 in various kinds of cancers. In all types of tumors, PSAT1 mutation rates were relatively low (less than 3%). As shown in Figure 3A, PSAT1 alterations are more frequently found in UCEC patients with a mutation than in skin cutaneous melanoma patients. The “amplification” and “deep deletion” type of CNA were the main type of sarcoma cancer cases and diffuse large B-cell Lymphoma cases, with an alteration frequency of ∼1.2 and ∼2.1% respectively. Figure 3B illustrates the types, locations, and number of genetically altered PSAT1 cases. The major type of genetic alteration was a missense mutation of PSAT1, with the highest alteration frequency being R213 H/C (Figure 3B). The 3D protein structure of PSAT1 was shown in Figure 3C.

Additionally, we examined whether alterations of PSAT1 genes affect different types of cancer patient’s prognosis. BRCA cases with altered PSAT1 showed a worse prognosis in DFS (*p* = 3.3e-09), disease-specific (*p* = 5.5e-06), OS (*p* = 2.1e-04) and PFS (*p* = 7.0e-11), compared with cases without PSAT1 alteration (Figure 3D).

The occurrence and development of tumors are closely related to RNA modification. Studies have shown that RNA modifying enzymes in tumor cells help to maintain cell proliferation and promote tumor progression. We analyzed the expression of PSAT1 and three kinds of RNA modified (10 m1A types, 13 m5C types, and 21 m6A types) marker genes in Pan cancer. Many cancer species, such as uveal melanoma (UVM), OC, UCEC, GBM, LUSC, etc., showed positive correlations between PSAT1 expression and RNA modification (Figure 3E).

### 3.4 Promoter methylation of PSAT1

**FIGURE 4:**
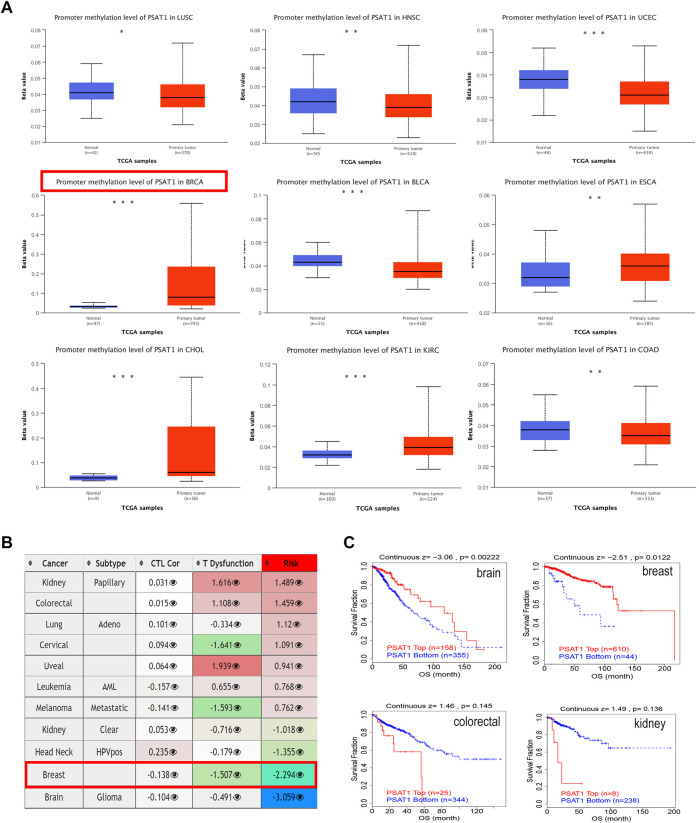
The methylation levels of PSAT1 in cancers and poor prognosis of cancer cohorts. **(A)** The methylation levels of PSAT1 between cancer and adjacent normal tissues through TCGA database. *p < 0.05; **p < 0.01; ***p < 0.001 **(B)** Heatmap showing the role of PSAT1 methylation in cytotoxic T-cell levels (CTLs), dysfunctional T-cell phenotype, and risk factors of TCGA cancer cohorts **(C)** Analyzing the difference in overall survival between PSAT1 hypermethylated and hypomethylated in various tumors through the TCGA database.

We studied the promoter methylation status of PSAT1 and found that this gene is highly methylated in BRCA, ESCA, CHOL, and KIRC. While it is hypomethylated in LUSC, HNSC, UCEC, BLCA and COAD (Figure 4A). Additionally, PSAT1 methylation status was studied in various cancer types and found to have a negative relationship with dysfunctional T cell phenotypes and shorter survival times of the breast cancer. Interestingly, although the methylation status of PSAT1 is not different between glioblastoma and normal tissues, hypermethylation of PSAT1 was associated with shorter survival duration of glioma (Figure 4B). Accordingly, we studied the effect of methylation status of PSAT1 on survival prognoses in breast cancer and glioblastoma, and found that hypermethylation status was associated with better outcomes (Figure 4C). In colorectal and kidney cancers, methylation status is not significantly correlated with prognosis, consistent with the result of Figure 4B.

### 3.5 Survival analysis data

**FIGURE 5:**
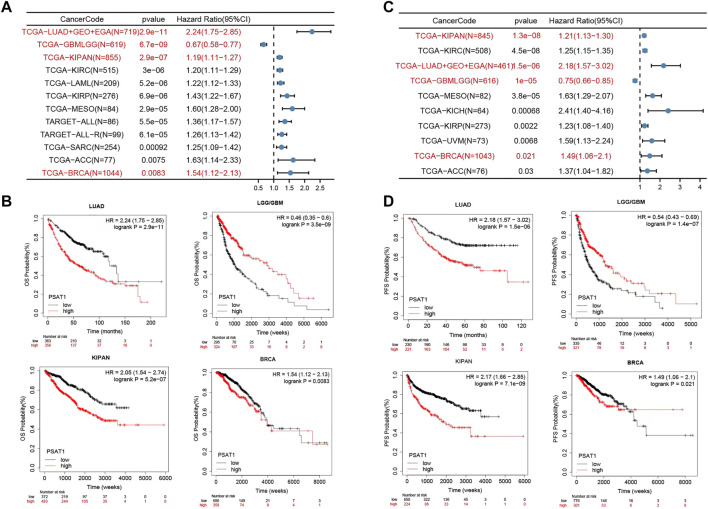
The relationship between PSAT1 gene expression and survival prognosis of cancers. **(A)** Forest map showing the relationship between the expression of PSAT1 and OS rate in Pan cancer **(B)** Kaplan-Meier curve showing the effect of PSAT1 gene expression on the OS in LUAD, LGG/GBM, KIPAN and BRCA **(C)** Forest map showing the relationship between the expression of PSAT1 and PFS rate in Pan cancer **(D)** Kaplan-Meier curve showing the effect of PSAT1 gene expression on the PFS in LUAD, LGG/GBM, KIPAN and BRCA.

For the purpose of assessing whether PSAT1 expression determines patients’ prognosis, we divided TCGA, Gene Expression Omnibus database (GEO) and European Genome-Phenome Archive (EGA) datasets based on the level of expression of PSAT1. According to Figure 5A, high expression of PSAT1 was associated with a poor OS prognosis in LUAD (*p* = 2.9e-11), KIPAN (*p* = 2.9e-07), KIRC (*P* = 3e-06), LAML (*p* = 5.2e-06), KIRP (*p* = 6.9e-06), Mesothelioma (MESO) (*p* = 2.9e-05) and BRCA (*p* = 8.3e-03) cancers. Nevertheless, high PSAT1 expression levels were associated with an improved OS prognosis in LGG and GBM (*p* = 6.7e-09) cancers.

Based on PFS analysis data (Figure 5C), high PSAT1 expression was associated with poor PFS in LUAD (*p* = 1.5e-06), KIPAN (*p* = 1.3e-08), KIRC (*p* = 4.5e-08), MESO (*p* = 3.8e-05), KICH (*p* = 6.8e-04), KIRP (*p* = 2.2e-03) and BRCA (*p* = 2.1e-02) cancers. However, high expression of the PSAT1 gene was related to better PFS prognosis in LGG and GBM (*p* = 1.0e-05). The relationship between the expression of PSAT1 in other cancer species and prognosis are shown in Supplementary Table S1.

According to the results of the above survival analysis, we found that the increased expression of PSAT1 was associated with poor prognosis of LUAD and BRCA (Figures 5B, D). Interestingly, the increased expression of PSAT1 is related to good prognosis of LGG and GBM, suggesting that PSAT1 may play different roles in different tumors.

### 3.6 Enrichment analysis of PSAT1 and related proteins

**FIGURE 6:**
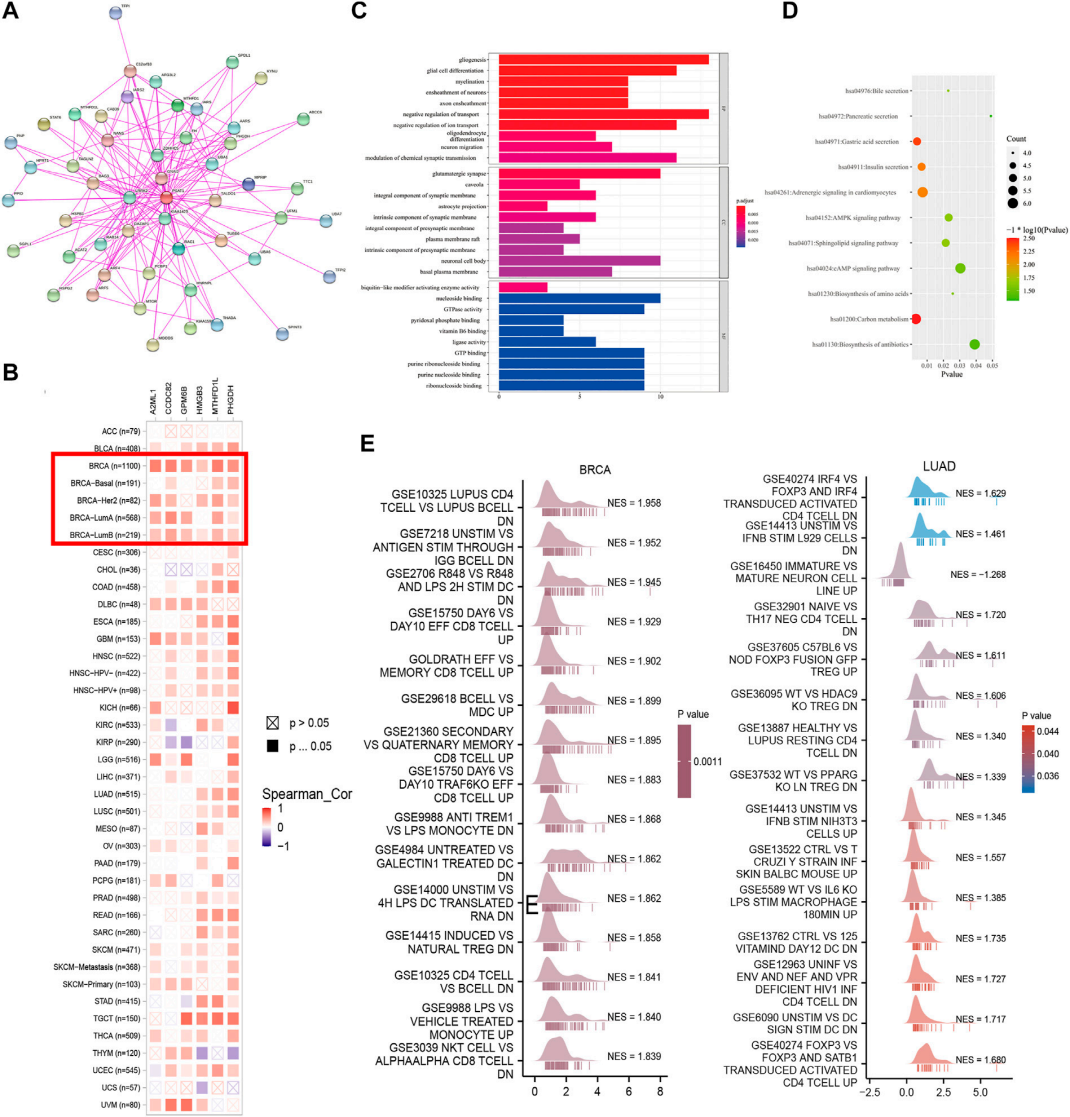
PSAT1-related gene enrichment analysis. **(A)** PSAT1-binding proteins were analyzed through an online tool String **(B)** The heatmap data of PSAT1 related genes in the TCGA project through GEPIA2 were displayed **(C)** KEGG pathway analysis was performed based on the PSAT1-binding and interacting genes **(D)** GO analysis for the molecular function. **(E)** GSEA analysis showing the enrichment of immune signal pathways in BRCA and LUAD.

In order to examine the molecular mechanisms involved in tumorigenesis and development of PSAT1, we screened out related genes that target PSAT1 binding protein and PSAT1 expression. Additionally, we performed a series of pathway enrichment analysis. In total, 50 PSAT1-binding proteins were identified using the STRING program, which was supported by experiments. Figure 6A is a gene interaction network diagram, which showed 50 binding proteins related to the PSAT1 gene. To determine which genes are associated with PSAT1 expression, all TCGA tumor expression data was evaluated using GEPIA2. A positive correlation was found between PSAT1 expression levels and expression levels of A2ML1 (*R* = 0.62), MTHFD1L (*R* = 0.63), PHGDH (*R* = 0.66), HMGB3 (*R* = 0.49) and GPM6B (*R* = 0.72) and CCDC82 (*R* = 0.64) genes (all *p* < 0.001) (Supplementary Figure S2). According to the heat map, PSAT1 is positively correlated with the above five genes in BRCA, indicating that these five genes may interact with PSAT1 in breast cancer (Figure 6B).

For the purpose of KEGG and Gene Ontology (GO) enrichment analysis, these two datasets were combined. According to Figure 6D, KEGG data indicated that PSAT1 may play a role in tumorigenesis through its effects on “carbon metabolism”, “acid secretion from the stomach”, and “adrenergic signaling in cardiomyocytes”. In GO enrichment analysis, molecular functional histogram showed that majority of these genes were associated with negative regulation of glial production, transport, glial cell differentiation and myelination sheathing (Figure 6C).

We further employed GSEA to investigate the potential biological processes of PSAT1 in pan-cancer according to the results of survival analysis. The results found that PSAT1 can be enriched into the classic signaling pathways of cancer such as mTORC1 signaling, MYC targets and JAK STAT3 signaling (Supplementary Figure S3). Further analysis showed that PSAT1 was enriched in immune related signaling pathways in LUAD and BRCA (Figure 6E).

### 3.7 Immune infiltration analysis data

**FIGURE 7:**
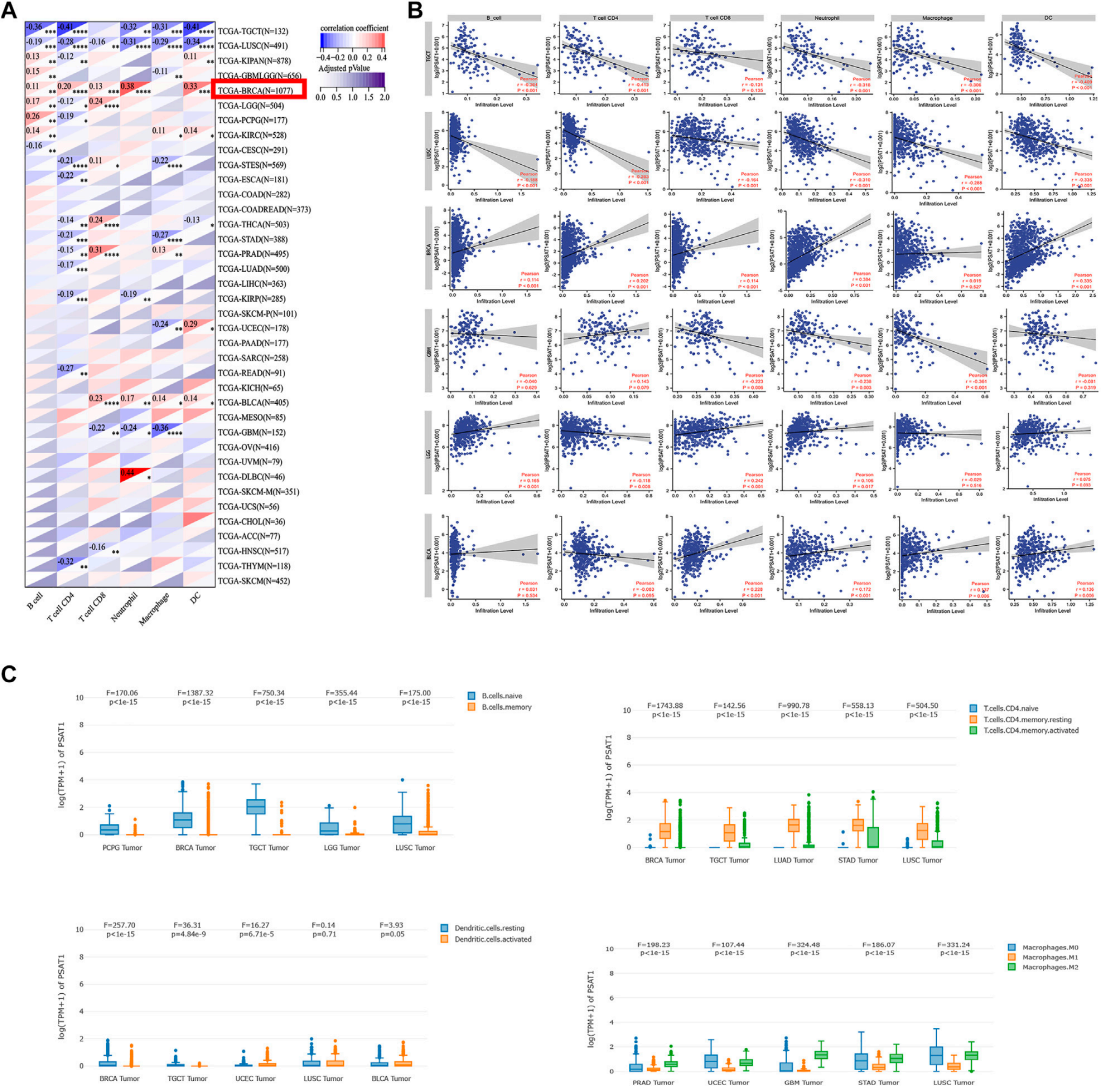
Correlation analysis between PSAT1 expression and immune infiltration of cancer-associated fibroblasts. **(A)** Heatmap showing correlation of PSAT1 expression with infiltration by six immune cell types, *p < 0.05; **p < 0.01; ***p < 0.001; ***p < 0.0001 **(B)** The detail relationship between the expression of PSAT1 and six immune cells infiltration in TGCT, LUSC,BRCA,GBM, LGG and BLCA.**(C)** The expression changes of immune cell subsets in different tumors.

To study the role of PSAT1 in the tumor immune microenvironment, we quantified its expression and immune infiltration levels in Pan-cancers. As seen in Figure 7A, PSAT1 expression is significantly correlated with the infiltrating of immune cells: B cells in 13 types of cancer, CD4^+^ T cells in 17 types of cancer, CD8^+^ T cells in 12 types of cancer, Neutrophil in 11 types of cancer, macrophages in 14 types of cancer and DCs in 13 types of cancer. We found the expression of PSAT1 is associated with immune cell infiltration in a number of tumor types, especially in BRCA and LUSC.

Based on the results in Figure 7A, we show in detail the relationship between PSAT1 expression and the infiltration of six immune cells, TGCT, LUSC, BRCA, GBM, LGG and BLCA, which have a greater correlation with immune cell infiltration (Figure 7B). TGCT and LUSC expressed PSAT1 in a significantly negative manner with regard to the infiltration of B cells, CD4^+^ T cells, macrophages, neutrophils, and DCs. Furthermore, PSAT1 in LUSC correlated negatively with CD8^+^ T cell infiltration as well. It was found that there was a significant correlation between PSAT1 expression and B-cell, CD4^+^ T-cell, CD8^+^ T-cell, neutrophil, and granulocyte infiltration in BRCA. BLCA revealed that PSAT1 expression was negatively correlated with the infiltration of CD8^+^ T cells, neutrophils, macrophages, and DCs. In GBM, PSAT1 expression showed a remarkable negative correlation with infiltration of CD8^+^ T cells, neutrophils, and macrophages. It is interesting to note that, as in glioma, LGG showed a clear positive correlation between PSAT1 expression and infiltration of B cells, CD8^+^ T cells and neutrophils, but a negatively correlation with infiltration of CD4^+^ T cells. This indicates that GBM and LGG are formed by different mechanisms and that PSAT1 plays a different role in GBM and LGG.

We further analyzed the changes in expression of immune cell subpopulations in different tumors based on the correlation between PSAT1 and immune cells. As shown in Figure 7C, memory B cells were lower than naive B cells in BRCA, TGCT and LUSC. Resting CD4^+^ T cells is higher than activated CD4^+^ T cells and naive CD4^+^ T cells in COAD, TGCT, BLCA, pheochromocytoma and paraganglioma (PCPG), GBM, UVM and BRCA. M2 macrophages is higher than M1 macrophages in BRCA, TGCT, LUSC, THCA, KIRC and GBM. Activated NK cell is higher than resting NK cell in THCA, HNSC and COAD. Based on the above research results between PSAT1 and the immune cells expression in cancers, PSAT1 is an important part of the immune microenvironment of Pan-cancer.

### 3.8 Pan-cancer analysis of immune modulators, TMB and MSI expression in relation to PSAT1 expression

**FIGURE 8:**
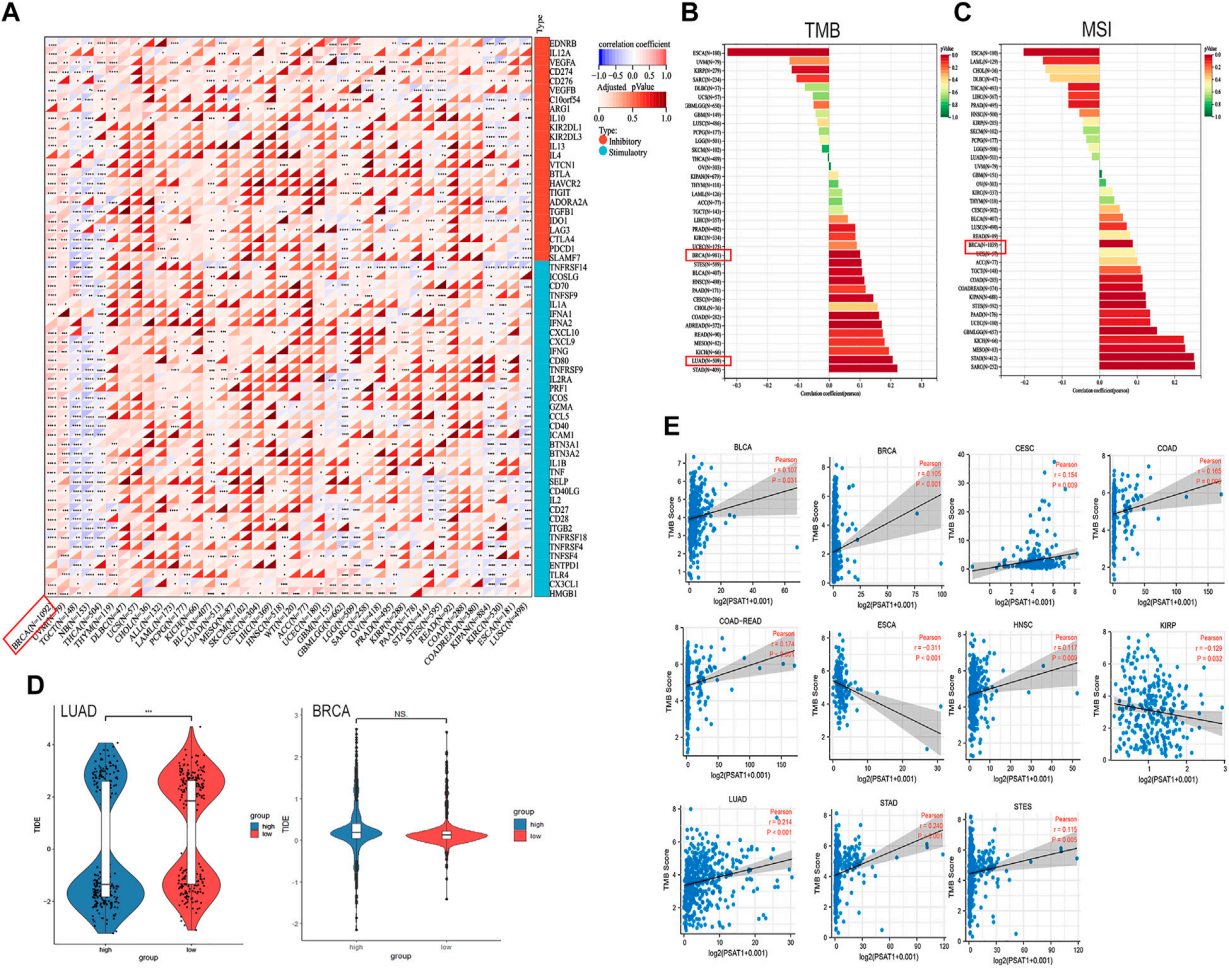
Correlation analyses of the PSAT1 expression with immune checkpoint genes, TMB, MSI and TIDE score. **(A)** Correlation analyses of the PSAT1 expression with immune checkpoint genes in pan-cancer. *p < 0.05 **(B)** A bar chart showing the association between PSAT1 expression and TMB in pan-cancer **(C)** A bar chart showing the association between PSAT1 gene expression and MSI in pan-cancer **(D)** Tide score in LUAD and BRCA, ***p < 0.001 **(E)** Cancer species with significant correlation between PSAT1 and TMB.

A tumor can utilize immune checkpoints to evade immune responses, including PD-1, PD-L1, and CTLA-4. Researchers need to investigate how PSAT1 expression is correlated with pan-cancer tumor microenvironment (TME), as well as examine how PSAT1 expression is correlated with two major immunomodulators. A Negative correlation between PSAT1 expression and most immunosuppressive and immunostimulatory agents was found in TGCT, Neuroblastoma (NB), KIPAN, LUSC and THCA (Figure 7A). It is worth mentioning that a positive correlation between PSAT1 expression and most immunosuppressive and immunostimulating agents has been observed in BRCA (Figure 8A).

Building on the correlation between PSAT1 and immune checkpoints, we took a step further to assess the therapeutic efficacy of immunotherapy for pan-cancer. TMB and MSI with PSAT1 expression was investigated, which are biomarkers of immunotherapeutic response. Moreover, TIDE score was also assessed. We found that expression of PSAT1 was correlated with both TMB and MSI in BRCA (Figures 8B, C). In addition, a remarkable correlation was found between PSAT1 expression and TMB in LUAD (Figure 8B), and the expression of PSAT1 was negatively correlated with the TIDE value (Figure 8D). Cancer species with significant correlation between PSAT1 and TMB are shown in Figure 7E. These results also together suggest that immunotherapy for LUAD and BRCA may be effective.

## 4 Discussion

Worldwide, tumors are currently the main threaten of health for people. Targeted therapies are a rising trend in cancer researches. The function of PSAT1 in diseases, including cancer, is being studied in ever-increasing numbers of studies. In certain cancer types, PSAT1 may contribute to the tumor initiation process, but this is unclear. Therefore, we analyzed in detail the role and possible mechanism of PSAT1 in Pan cancers. This study combined gene expression, gene mutation, methylation, immune microenvironment, pathway enrichment, and survival prognosis to comprehensively detect the expression of PSAT1 gene in different cancers on account of data from the TCGA, GEO, and CPTAC databases.

In the second step of serine synthesis, PSAT1 is an important transaminase that links glycolysis and serine biosynthesis and 3-phosphate hydroxypyruvate was converted to phosphoserine. Purine nucleotides, phosphatidylcholine, phosphatidylserine and other cellular metabolites are serine dependent as a carbon source. Therefore, PSAT1 is also an important substance in the metabolic pathway of cancer cells. Former studies have illustrated that serine biosynthesis has a key role in bone metastatic breast cancer, and three enzymes, PSAT1, phosphoserine phosphatase and phosphoglycerol dehydrogenase, are in charge of the phosphorylation pathway of L-serine biosynthesis (Pollari et al., 2011). Depending on the tumor type, several reports have implicated PSAT1 in many oncogenic processes including proliferation, migration, invasion, and chemoresistance (Vié et al., 2008; Yang et al., 2015; Kottakis et al., 2016; Liu et al., 2016; De Marchi et al., 2017; Gao et al., 2017; Dai et al., 2019; Metcalf et al., 2020). Our results also suggested that PSAT1 is highly expressed in many malignant tumors and is associated with advanced stage of tumors. At the same time, high expression of PSAT1 is associated with poor prognosis in many tumors. The role of PSAT1 in some malignant tumors has been explored initially (Ojala et al., 2002; Vié et al., 2008). Based on loss of function and gain of function experiments, PSAT1 is generally shown to promote cell cycle progression, proliferation, and tumorigenesis (Yang et al., 2015). There is some evidence that PSAT1 expression is related to malignant metastasis, chemotherapy sensitivity, and poor prognoses (Vié et al., 2008). In colorectal cancers, PSAT1 is considered the most upregulated gene and its overexpression is linked to advanced tumor stage, chemo-resistance and poor prognosis with endocrine therapy (Qian et al., 2017). Our pan cancer analysis also found this result. The expression of PSAT1 was significantly increased in colorectal cancer. In non-small cell lung cancer (NSCLC), PSAT1 has been reported to be significantly increased. This gene may also be involved in proliferation and cell cycle regulation of tumor cells (Yang et al., 2015). PSAT1 potentiates G1 activity by modulating the degradation of cyclin D1 and the activity of the Rb-E2F pathway, indicating its unique intracellular signaling axis. Patients with NSCLC with elevated PSAT1 expression have a poor clinical prognosis. This is consistent with our research results that our findings also showed that the expression of PSAT1 in lung cancer tissues was higher than that in normal tissues. In addition, Stephanie Metcalf et al. demonstrated that the expression of PSAT1 in triple negative breast cancer was higher than that in normal tissues, and increased with the increase of breast cancer stage (Metcalf et al., 2020). Interestingly, our results showed that PSAT1 first increased and then decreased with the increase of breast cancer stage, and its expression in breast cancer was lower than that in normal tissues. This may be because we did not classify breast cancer for subgroup analysis. Song Gao et al. also found for the first time that the expression of PSAT1 was significantly upregulated in ER-negative breast cancers compared with ER-positive breast cancers (Gao et al., 2017). This also reflects that our research results are inconsistent with the previous results, which may be due to different mechanism of PSAT1 in different pathological types of breast cancer.

It is worth mentioning that Stanley Ching et al. found that PERK activation mediates the up regulation of PSAT1 and serine biosynthesis through the downstream transcription factor ATF-4, and PERK is the key metabolic hub of macrophage immunosuppressive function (Raines et al., 2022). Their results describe for the first time the relationship between PERK signaling and PSAT1 mediated serine metabolism, and also suggest that PSAT1 is involved in the regulation of tumor immune microenvironment. This is consistent with our research that we found PSAT1 may be an important part of the immune microenvironment of BRCA and LUAD, and it may be a potential biomarker to predict the efficacy of immunotherapy for BRCA and LUAD.

Our research found that the expression level of PSAT1 in BLCA, CESC, COAD, ESCA, HNSC, KICH, LUAD, PRAD, READ, STAD, UCEC and LUSC tumor tissues was higher than the corresponding control tissues. However, the expression is lower in BRCA, CHOL, KIRC, KIRP, liver hepatocellular carcinoma (LIHC) and THCA. This difference in expression levels may reflect the differences in the function and mechanism of PSAT1 in different tumors. Our further analysis found that for tumor patients with higher PSAT1 expression, such as ACC, KIRC, KIRP, LIHC, sarcoma (SARC) and UVM, the overexpression of PSAT1 is associated with poor OS. According to these findings, PSAT1 is a potential biomarker for cancer prognosis prediction. We noted that the increase in PSAT1 was associated with poor prognosis of survival in most tumors. However, it is rare to find PSAT1 in mesothelioma in previous studies. We may have discovered a new biomarker that predicting the survival of patients with mesothelioma.

In our analysis, several noteworthy findings were found, especially in breast cancer. The results revealed that PSAT1 expression was varied in different stages of breast cancer. Its expression increases in breast cancer stages I to II, but in stages III to X, the expression of PSAT1 gradually decreases. This suggests that elevated PSAT1 in the early stage of breast cancer may be one of the markers for metastasis. However, Further analysis revealed that patients with elevated PSAT1 expression had a better prognosis. Hence, the prognostic significance of PSAT1 needs to be dynamically evaluated at different stages of breast cancer. In addition, molecular experimental studies must be conducted in order to determine if PSAT1 expression is related to cancer development in the above tumor types. We found that BRCA cases with altered PSAT1 showed worse prognosis in DFS, OS and PFS, compared with cases without PSAT1 alteration.

Interruption, progression, and metastasis of cancer are closely linked to the actions of tumor-infiltrating immune cells, which are prominent components in the tumor microenvironment (Fridman et al., 2010; Steven and Seliger, 2018). Researchers reported that stromal fibroblasts contribute to the modulation of tumor-infiltrating immune cells by modulating the function of cancer-associated fibroblasts (Chen and Song, 2019; Kwa et al., 2019). In light of this, examining the relationship between PSAT1 expression and immune infiltration is of great importance. Among the main findings of our study is the association between PSAT1 expression and immune infiltration in a variety of cancers, especially BRCA, Luminal, and LGG. Our results have demonstrated that PSAT1 expression responded positively to the level of infiltration of B cells, CD8^+^ T cells, CD4^+^ T cells, macrophages, neutrophils and DCs in BRCA Luminal and LGG. Interestingly, the expression level of PSAT1 in LGG has nothing to do with tumor purity, which indicates that it has the same expression in tumor cells and tumor microenvironment. The PSAT1 expression level in BRCA Luminal, however, has a significant negative correlation with tumor purity, indicating enhanced expression within the tumor microenvironment. B cells and macrophages are important antigen-presenting cells. PSAT1 expression is significantly associated with B cell and macrophage expression in BRCA Luminal, GBM, and LGG. In both TGCT and THCA, however, B cells and macrophages were negatively correlated with PSAT1. These variations suggest that tumors are somewhat heterogeneous in their recruitment of antigen-presenting cells to TME. In conclusion, these results demonstrate that PSAT1 plays a significant role in the recruitment and the modulation of tumor immune infiltrating cells and may ultimately have an impact on patients’ survival.

In our study, the prognostic value of PSAT1 in pan-cancer was confirmed. It was found that PSAT1 with high expression in KIRP, KIRC and MESO had worse OS and DFS comparing with low expression. On the contrary, high PSAT1 expression levels are associated with better OS and DFS of LGG and GBM. This indicates that PSAT1 may play a completely different role in glioma compared to other tumors, which also reflects the heterogeneity of tumor occurrence and development and the particularity of the brain tumor microenvironment. Interestingly, our results found that in STAD patients with high expression of PSAT1, the duration of DFS was longer, but there was no significant difference in OS. This may be because the tumor in STAD patients progresses quickly after recurrence, and the specific cause needs further clinical and basic research to determine.

However, although we integrated information from multiple databases and came up with some meaningful results, this study still has limitations. Firstly, the results of this study mainly come from the data analysis and bioinformatics analysis of the public database website. It is preliminarily concluded that PSAT1 may play an immune related role in lung cancer and breast cancer, and has certain predictability for the efficacy of immunotherapy. However, we have no corresponding basic research validation and clinical patient data validation, further studies on the mechanisms of PSAT1 at the cellular and molecular level will help to elucidate the role of PSAT1. Secondly, we still cannot define PSAT1 as a friend or an enemy, because from different databases, we found some conflicting results. To clarify this point requires further and more in-depth mechanism research. Thirdly, while PSAT1 expression was found to be involved in immune cell infiltration of cancer, we could not demonstrate that PSAT1 affects patient survival through immune infiltration. Further prospective clinical studies may address this question.

## 5 Conclusion

Our first pan-cancer analysis of PSAT1 has identified statistically significant correlations between the expression of PSAT1 and clinical prognosis, DNA methylation, gene mutations, cellular immunological response, etc. Facilitate the clarification of the role of PSAT1 in tumorigenesis from diverse perspectives. PSAT1 may influence pan-cancer prognosis and is associated with immune infiltration, particularly for BRCA and LUSC. PSAT1 may be a new biomarker for predicting the efficacy of immunotherapy for lung cancer and breast cancer. These findings possibly provide a mechanism for manoeuvring the energetic system of tumor cell or tumor microenvironment infiltration to counteract tumor growth.

## Data Availability

The original contributions presented in the study are included in the article/Supplementary Material, further inquiries can be directed to the corresponding authors.
