# Supplementary figures and images for: An integrated pan-cancer analysis of PSAT1: A potential biomarker for survival and immunotherapy
Source: Front Genet. 2022 Aug 29;13:975381. doi: 10.3389/fgene.2022.975381 (PMC9465327; doi:10.3389/fgene.2022.975381)

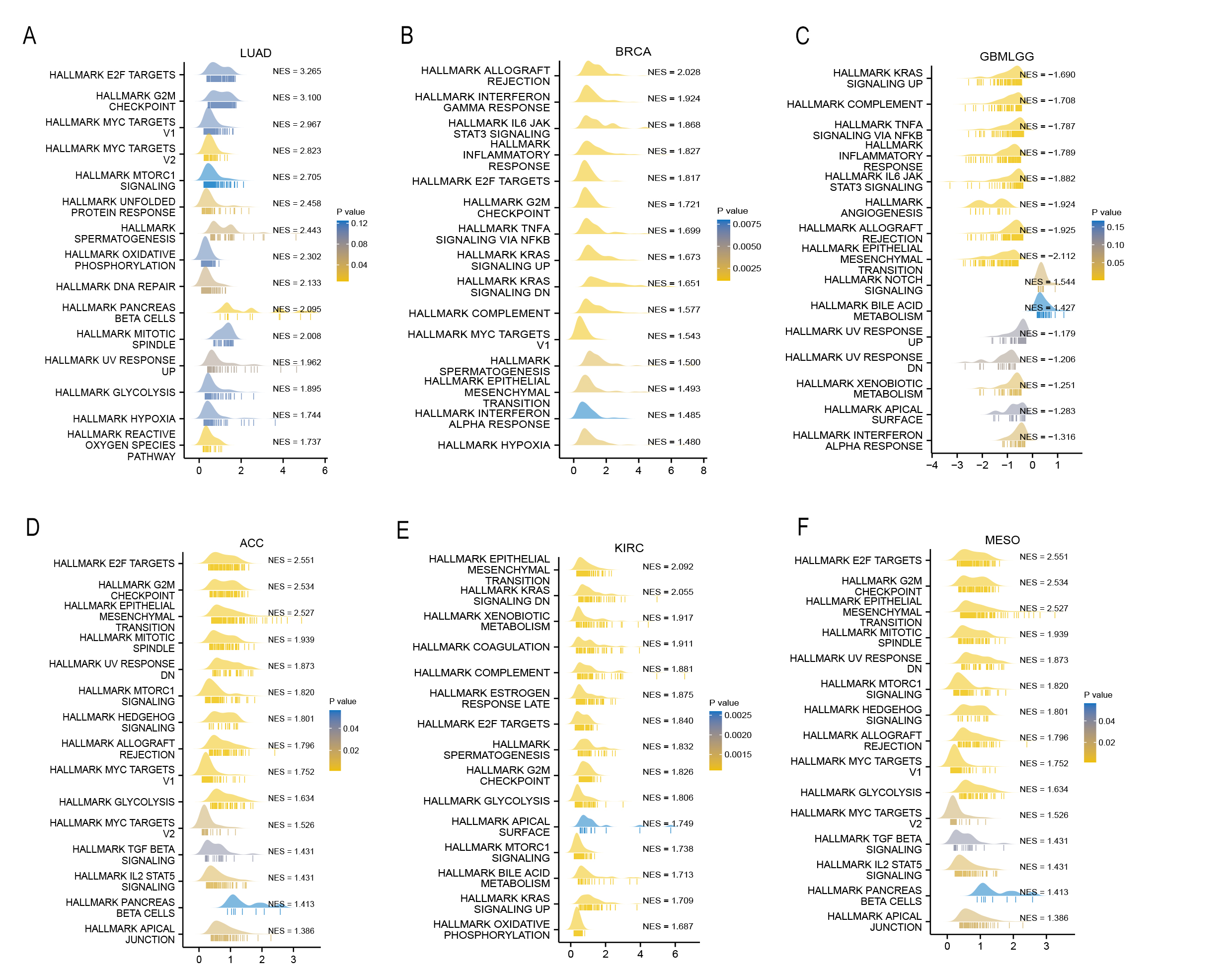

Supplement: Supplementary file 1 [file Image3.JPEG]

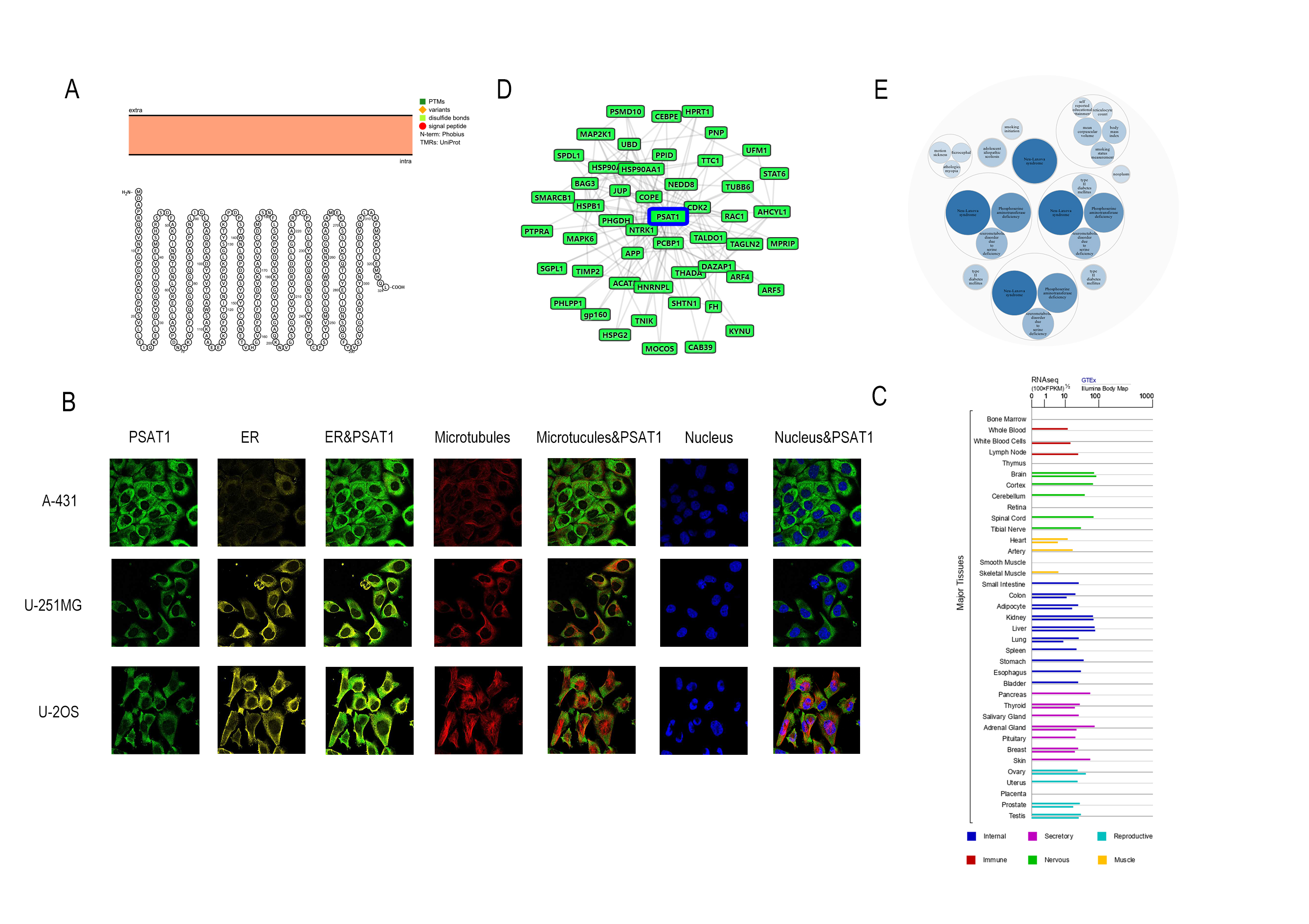

Supplement: Supplementary file 3 [file Image1.JPEG]

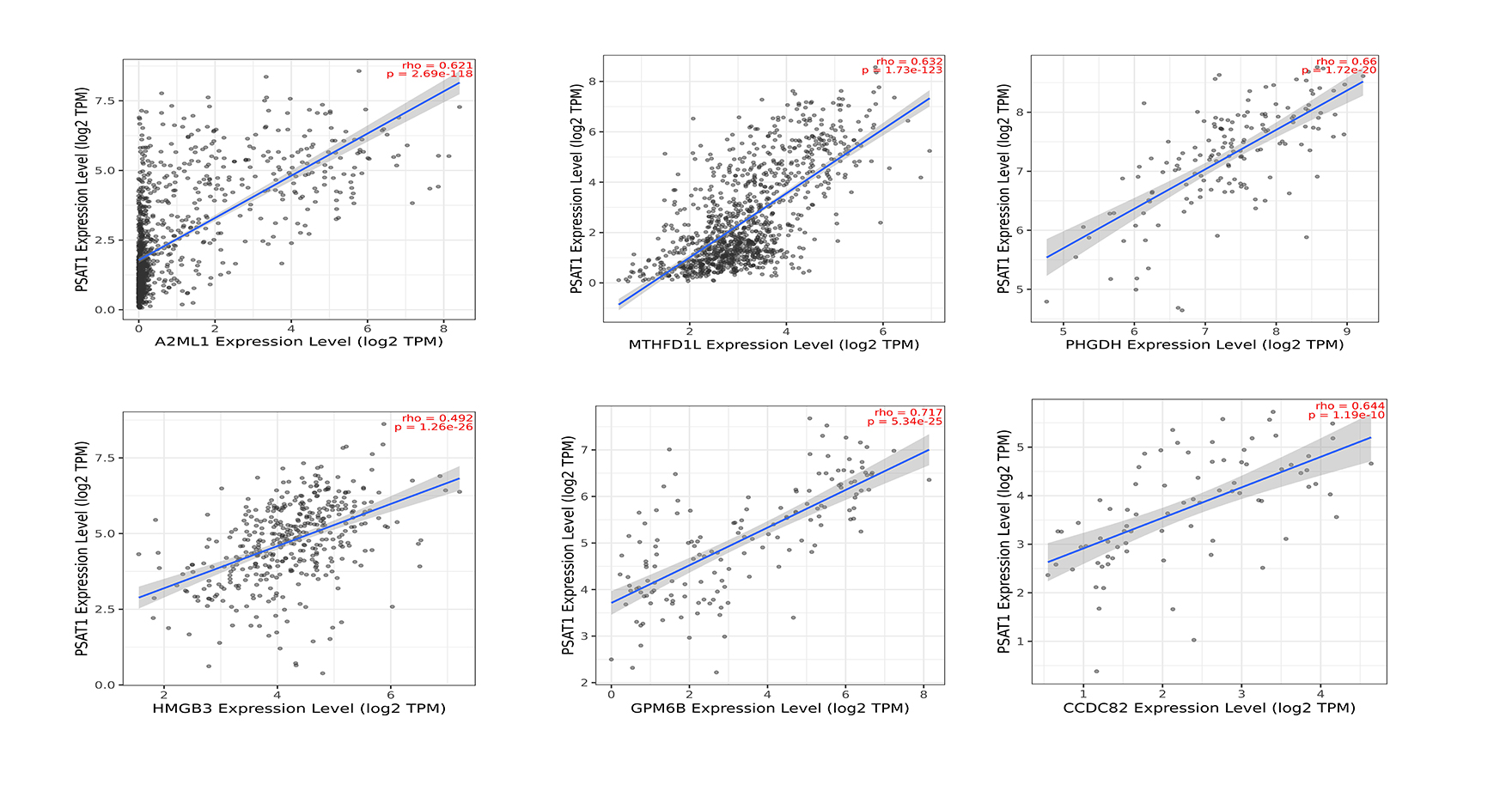

Supplement: Supplementary file 4 [file Image2.JPEG]
