# Supplementary material for: An integrated pan-cancer analysis of PSAT1: A potential biomarker for survival and immunotherapy
Source: Front Genet. 2022 Aug 29;13:975381. doi: 10.3389/fgene.2022.975381 (PMC9465327; doi:10.3389/fgene.2022.975381)
Supplement: Supplementary file 2 [file Table1.DOCX]

Table1 The relationship between expression of PSAT1 and OS in Pan cancer

| Cancer Code | P value | Hazard Ratio(95%CI) |
| --- | --- | --- |
| \| TCGA-LUAD+GEO+EGA(N=719) \| \| --- \| \| TCGA-GBMLGG(N=619) \| \| TCGA-KIPAN(N=855) \| \| TCGA-KIRC(N=515) \| \| TCGA-LAML(N=209) \| \| TCGA-KIRP(N=276) \| \| TCGA-MESO(N=84) \| \| TARGET-ALL(N=86) \| \| TARGET-ALL-R(N=99) \| \| TCGA-SARC(N=254) \| \| TCGA-ACC(N=77) \| \| TCGA-BRCA(N=1879) \| \| TCGA-KICH(N=64) \| \| TCGA-THCA(N=501) \| \| TARGET-NB(N=151) \| \| TCGA-UVM(N=74) \| \| TCGA-LIHC(N=341) \| \| TCGA-LGG(N=474) \| \| TCGA-PCPG(N=170) \| \| TARGET-LAML(N=142) \| \| TCGA-THYM(N=117) \| \| TCGA-READ(N=90) \| \| TCGA-PRAD(N=492) \| \| TCGA-STAD+GEO+EGA(N=875) \| \| TCGA-BLCA(N=398) \| \| TCGA-UCS(N=55) \| \| TCGA-HNSC(N=509) \| \| TCGA-OV+GEO+EGA(N=1656) \| \| TCGA-PAAD(N=172) \| \| TCGA-GBM(N=144) \| \| TARGET-WT(N=80) \| \| TCGA-STES(N=547) \| \| TCGA-COADREAD(N=368) \| \| TCGA-UCEC(N=166) \| \| TCGA-DLBC(N=44) \| \| TCGA-CHOL(N=33) \| \| TCGA-ESCA(N=175) \| \| TCGA-LUSC+GEO+EGA(N=524) \| \| TCGA-SKCM-P(N=97) \| \| TCGA-TGCT(N=128) \| \| TCGA-CESC(N=273) \| \| TCGA-SKCM(N=444) \| \| TCGA-SKCM-M(N=347) \| \| TCGA-COAD(N=278) \| | \| 2.90E-11 \| \| --- \| \| 6.70E-09 \| \| 2.90E-07 \| \| 3.00E-06 \| \| 5.20E-06 \| \| 6.90E-06 \| \| 2.90E-05 \| \| 5.50E-05 \| \| 6.10E-05 \| \| 9.20E-04 \| \| 7.50E-03 \| \| 8.30E-03 \| \| 3.00E-02 \| \| 6.00E-02 \| \| 6.00E-02 \| \| 7.00E-02 \| \| 1.00E-01 \| \| 1.00E-01 \| \| 1.60E-01 \| \| 1.80E-01 \| \| 2.10E-01 \| \| 2.10E-01 \| \| 2.50E-01 \| \| 2.60E-01 \| \| 2.70E-01 \| \| 3.00E-01 \| \| 3.20E-01 \| \| 4.10E-01 \| \| 4.40E-01 \| \| 4.60E-01 \| \| 5.80E-01 \| \| 5.90E-01 \| \| 6.00E-01 \| \| 6.10E-01 \| \| 6.50E-01 \| \| 7.40E-01 \| \| 7.90E-01 \| \| 8.00E-01 \| \| 8.10E-01 \| \| 8.30E-01 \| \| 8.70E-01 \| \| 9.00E-01 \| \| 9.30E-01 \| \| 9.70E-01 \| | \| 2.24(1.75-2.85) \| \| --- \| \| 0.67(0.58-0.77) \| \| 1.19(1.11-1.27) \| \| 1.20(1.11-1.29) \| \| 1.22(1.12-1.33) \| \| 1.43(1.22-1.67) \| \| 1.60(1.28-2.00) \| \| 1.36(1.17-1.57) \| \| 1.26(1.13-1.42) \| \| 1.25(1.09-1.42) \| \| 1.63(1.14-2.33) \| \| 1.54(1.12-2.13) \| \| 1.74(1.04-2.92) \| \| 1.27(0.99-1.64) \| \| 1.25(0.99-1.59) \| \| 1.39(0.98-1.99) \| \| 1.11(0.98-1.25) \| \| 0.83(0.66-1.04) \| \| 1.73(0.81-3.70) \| \| 1.06(0.98-1.14) \| \| 1.41(0.82-2.41) \| \| 0.80(0.56-1.14) \| \| 1.55(0.74-3.27) \| \| 0.90(0.76-1.08) \| \| 1.07(0.95-1.21) \| \| 1.19(0.85-1.66) \| \| 1.06(0.94-1.20) \| \| 0.95(0.83-1.08) \| \| 1.06(0.91-1.24) \| \| 0.93(0.77-1.13) \| \| 0.88(0.56-1.38) \| \| 0.97(0.89-1.07) \| \| 0.95(0.80-1.14) \| \| 1.06(0.84-1.33) \| \| 1.11(0.71-1.72) \| \| 1.04(0.83-1.30) \| \| 1.03(0.83-1.29) \| \| 1.03(0.81-1.31) \| \| 1.02(0.86-1.21) \| \| 1.11(0.43-2.88) \| \| 0.98(0.81-1.20) \| \| 1.00(0.95-1.06) \| \| 1.00(0.94-1.06) \| \| 1.00(0.81-1.24) \| |

Note: p < 0.05 was considered to be significant.

Abbreviations: OS, overall survival.

Table2 The relationship between expression of PSAT1 and PFS in Pan cancer

| Cancer Code | P value | Hazard Ratio(95%CI) |
| --- | --- | --- |
| \| TCGA-KIPAN(N=845) \| \| --- \| \| TCGA-KIRC(N=508) \| \| TCGA-LUAD+GEO+EGA(N=461) \| \| TCGA-GBMLGG(N=616) \| \| TCGA-MESO(N=82) \| \| TCGA-KICH(N=64) \| \| TCGA-KIRP(N=273) \| \| TCGA-UVM(N=73) \| \| TCGA-BRCA(N=1043) \| \| TCGA-ACC(N=76) \| \| TCGA-PAAD(N=171) \| \| TCGA-TGCT(N=126) \| \| TCGA-BLCA(N=397) \| \| TCGA-DLBC(N=43) \| \| TCGA-HNSC(N=508) \| \| TCGA-GBM(N=143) \| \| TCGA-LUSC+GEO+EGA(N=141) \| \| TCGA-OV+GEO+EGA(N=1435) \| \| TCGA-READ(N=88) \| \| TCGA-SKCM(N=434) \| \| TCGA-SKCM-M(N=338) \| \| TCGA-UCS(N=55) \| \| TCGA-LGG(N=472) \| \| TCGA-LIHC(N=340) \| \| TCGA-STAD+GEO+EGA(N=640) \| \| TCGA-COADREAD(N=363) \| \| TCGA-PCPG(N=168) \| \| TCGA-STES(N=548) \| \| TCGA-THCA(N=499) \| \| TCGA-SARC(N=250) \| \| TCGA-THYM(N=117) \| \| TCGA-SKCM-P(N=96) \| \| TCGA-CESC(N=273) \| \| TCGA-COAD(N=275) \| \| TCGA-CHOL(N=33) \| \| TCGA-UCEC(N=166) \| \| TCGA-PRAD(N=492) \| \| TCGA-ESCA(N=173) \| | \| 1.30E-08 \| \| --- \| \| 4.50E-08 \| \| 1.50E-06 \| \| 1.00E-05 \| \| 3.80E-05 \| \| 6.80E-04 \| \| 2.20E-03 \| \| 6.80E-03 \| \| 0.021 \| \| 0.03 \| \| 0.09 \| \| 0.09 \| \| 0.15 \| \| 0.19 \| \| 0.25 \| \| 0.25 \| \| 0.28 \| \| 0.31 \| \| 0.33 \| \| 0.34 \| \| 0.37 \| \| 0.38 \| \| 0.42 \| \| 0.44 \| \| 0.46 \| \| 0.51 \| \| 0.55 \| \| 0.58 \| \| 0.59 \| \| 0.6 \| \| 0.68 \| \| 0.68 \| \| 0.71 \| \| 0.72 \| \| 0.76 \| \| 0.87 \| \| 0.92 \| \| 0.94 \| | \| 1.21(1.13-1.30) \| \| --- \| \| 1.25(1.15-1.35) \| \| 2.18(1.57-3.02) \| \| 0.75(0.66-0.85) \| \| 1.63(1.29-2.07) \| \| 2.41(1.40-4.16) \| \| 1.23(1.08-1.40) \| \| 1.59(1.13-2.24) \| \| 1.49(1.06-2.1) \| \| 1.37(1.04-1.82) \| \| 1.14(0.98-1.32) \| \| 1.30(0.96-1.75) \| \| 1.10(0.97-1.24) \| \| 0.84(0.65-1.09) \| \| 1.08(0.95-1.22) \| \| 0.90(0.75-1.08) \| \| 1.33(0.85-2.22) \| \| 0.93(0.83-1.07) \| \| 0.85(0.61-1.18) \| \| 0.98(0.93-1.02) \| \| 0.98(0.93-1.03) \| \| 1.15(0.84-1.58) \| \| 0.93(0.77-1.12) \| \| 1.04(0.94-1.15) \| \| 1.08(0.88-1.32) \| \| 0.95(0.80-1.12) \| \| 1.12(0.77-1.62) \| \| 0.97(0.89-1.07) \| \| 0.96(0.81-1.13) \| \| 1.03(0.93-1.14) \| \| 0.94(0.70-1.25) \| \| 0.97(0.85-1.11) \| \| 0.96(0.80-1.17) \| \| 0.96(0.79-1.17) \| \| 0.97(0.81-1.17) \| \| 0.98(0.82-1.19) \| \| 1.01(0.81-1.26) \| \| 1.01(0.82-1.24) \| |

Note: p < 0.05 was considered to be significant.

Abbreviations: PFS, Progression-Free-Survival.
