# Supplementary material for: An integrated pan-cancer analysis of PSAT1: A potential biomarker for survival and immunotherapy
Source: Front Genet. 2022 Aug 29;13:975381. doi: 10.3389/fgene.2022.975381 (PMC9465327; doi:10.3389/fgene.2022.975381)
Supplement: Supplementary file 5 [file Table2.DOCX]

Table2 The subtypes of BRCA and immune subtypes

| TCGA Participant Barcode | TCGA Study | Immune Subtype | TCGA Subtype |
| --- | --- | --- | --- |
| TCGA-4H-AAAK | BRCA | C1 | BRCA.LumA |
| TCGA-A1-A0SM | BRCA | C1 | BRCA.LumA |
| TCGA-A2-A0CO | BRCA | C2 | BRCA.LumA |
| TCGA-A2-A0CQ | BRCA | C4 | BRCA.LumA |
| TCGA-A2-A0EU | BRCA | C1 | BRCA.LumA |
| TCGA-A2-A0SX | BRCA | C2 | BRCA.Basal |
| TCGA-A2-A0SY | BRCA | C3 | BRCA.LumA |
| TCGA-A2-A0T2 | BRCA | C1 | BRCA.Basal |
| TCGA-A2-A0T5 | BRCA | C1 | BRCA.LumA |
| TCGA-A2-A0YK | BRCA | C3 | BRCA.Normal |
| TCGA-A2-A1G4 | BRCA | C1 | BRCA.LumB |
| TCGA-A7-A6VY | BRCA | C2 | BRCA.Basal |
| TCGA-A8-A06N | BRCA | C4 | BRCA.LumB |
| TCGA-A8-A07C | BRCA | C2 | BRCA.Basal |
| TCGA-A8-A081 | BRCA | C2 | BRCA.LumB |
| TCGA-A8-A08A | BRCA | C3 | BRCA.LumA |
| TCGA-A8-A08B | BRCA | C2 | BRCA.Her2 |
| TCGA-A8-A08O | BRCA | C1 | BRCA.LumA |
| TCGA-A8-A099 | BRCA | C3 | BRCA.LumA |
| TCGA-A8-A09X | BRCA | C2 | BRCA.Her2 |
| TCGA-A8-A0A2 | BRCA | C3 | BRCA.LumA |
| TCGA-AC-A2B8 | BRCA | C6 | BRCA.LumA |
| TCGA-AC-A2QI | BRCA | C3 | BRCA.LumA |
| TCGA-AC-A3W7 | BRCA | C3 | BRCA.LumA |
| TCGA-AC-A62X | BRCA | C2 | BRCA.Basal |
| TCGA-AN-A0FL | BRCA | C2 | BRCA.Basal |
| TCGA-AN-A0G0 | BRCA | C1 | BRCA.Basal |
| TCGA-AN-A0XV | BRCA | C1 | BRCA.LumA |
| TCGA-AO-A0J8 | BRCA | C2 | BRCA.LumA |
| TCGA-AO-A0JA | BRCA | C1 | BRCA.LumA |
| TCGA-AQ-A04J | BRCA | C1 | BRCA.Basal |
| TCGA-AR-A0TQ | BRCA | C2 | BRCA.LumB |
| TCGA-AR-A1AK | BRCA | C2 | BRCA.LumA |
| TCGA-AR-A1AP | BRCA | C2 | BRCA.LumA |
| TCGA-AR-A1AY | BRCA | C1 | BRCA.Basal |
| TCGA-AR-A24R | BRCA | C2 | BRCA.LumB |
| TCGA-AR-A252 | BRCA | C2 | BRCA.LumA |
| TCGA-AR-A2LE | BRCA | C3 | BRCA.LumA |
| TCGA-AR-A5QM | BRCA | C6 | BRCA.LumA |
| TCGA-B6-A0IB | BRCA | C1 | BRCA.LumB |
| TCGA-B6-A0WZ | BRCA | C2 | BRCA.LumA |
| TCGA-B6-A0X1 | BRCA | C2 | BRCA.Basal |
| TCGA-B6-A401 | BRCA | C1 | BRCA.LumA |
| TCGA-BH-A0B8 | BRCA | C1 | BRCA.Normal |
| TCGA-BH-A0B9 | BRCA | C2 | BRCA.Basal |
| TCGA-BH-A0BT | BRCA | C2 | BRCA.Normal |
| TCGA-BH-A0C0 | BRCA | C2 | BRCA.Normal |
| TCGA-BH-A0DE | BRCA | C6 | BRCA.LumA |
| TCGA-BH-A0DH | BRCA | C1 | BRCA.Normal |
| TCGA-BH-A0DT | BRCA | C3 | BRCA.Normal |
| TCGA-BH-A0DV | BRCA | C3 | BRCA.Normal |
| TCGA-BH-A0EE | BRCA | C2 | BRCA.Her2 |
| TCGA-BH-A0H5 | BRCA | C6 | BRCA.Normal |
| TCGA-BH-A0HO | BRCA | C4 | BRCA.LumA |
| TCGA-BH-A0HY | BRCA | C1 | BRCA.LumB |
| TCGA-BH-A18Q | BRCA | C2 | BRCA.Normal |
| TCGA-BH-A18U | BRCA | C2 | BRCA.Normal |
| TCGA-BH-A1EN | BRCA | C4 | BRCA.Normal |
| TCGA-BH-A1EO | BRCA | C6 | BRCA.LumA |
| TCGA-BH-A1F6 | BRCA | C2 | BRCA.Normal |
| TCGA-BH-A1FU | BRCA | C1 | BRCA.Normal |
| TCGA-BH-A42T | BRCA | C1 | BRCA.LumB |
| TCGA-BH-A6R9 | BRCA | C1 | BRCA.Normal |
| TCGA-BH-A8FY | BRCA | C4 | BRCA.LumA |
| TCGA-C8-A137 | BRCA | C1 | BRCA.Her2 |
| TCGA-C8-A1HG | BRCA | C2 | BRCA.LumB |
| TCGA-C8-A1HN | BRCA | C4 | BRCA.LumB |
| TCGA-C8-A8HR | BRCA | C2 | BRCA.Normal |
| TCGA-D8-A1XS | BRCA | C1 | BRCA.LumB |
| TCGA-D8-A1Y1 | BRCA | C4 | BRCA.LumB |
| TCGA-D8-A27N | BRCA | C2 | BRCA.LumB |
| TCGA-D8-A73U | BRCA | C3 | BRCA.LumA |
| TCGA-D8-A73X | BRCA | C3 | BRCA.LumA |
| TCGA-E2-A14S | BRCA | C4 | BRCA.LumB |
| TCGA-E2-A1IE | BRCA | C1 | BRCA.LumA |
| TCGA-E2-A1IJ | BRCA | C3 | BRCA.LumA |
| TCGA-E2-A1IL | BRCA | C2 | BRCA.LumA |
| TCGA-E2-A1L7 | BRCA | C2 | BRCA.Normal |
| TCGA-E9-A1NF | BRCA | C1 | BRCA.LumB |
| TCGA-E9-A1RA | BRCA | C1 | BRCA.LumA |
| TCGA-E9-A229 | BRCA | C2 | BRCA.LumA |
| TCGA-E9-A248 | BRCA | C2 | BRCA.Her2 |
| TCGA-E9-A5FL | BRCA | C2 | BRCA.Basal |
| TCGA-E9-A6HE | BRCA | C2 | BRCA.LumA |
| TCGA-EW-A1OY | BRCA | C2 | BRCA.LumB |
| TCGA-EW-A1P3 | BRCA | C1 | BRCA.LumA |
| TCGA-EW-A1PC | BRCA | C2 | BRCA.LumB |
| TCGA-EW-A2FR | BRCA | C1 | BRCA.Her2 |
| TCGA-EW-A6S9 | BRCA | C2 | BRCA.LumA |
| TCGA-GM-A2DA | BRCA | C1 | BRCA.LumA |
| TCGA-GM-A2DH | BRCA | C2 | BRCA.Her2 |
| TCGA-GM-A2DL | BRCA | C1 | BRCA.LumA |
| TCGA-GM-A5PV | BRCA | C3 | BRCA.LumA |
| TCGA-LL-A5YO | BRCA | C2 | BRCA.Her2 |
| TCGA-LL-A5YP | BRCA | C1 | BRCA.Basal |
| TCGA-OL-A5RU | BRCA | C3 | BRCA.LumA |
| TCGA-S3-AA15 | BRCA | C1 | BRCA.Basal |
| TCGA-A1-A0SD | BRCA | C2 | BRCA.LumA |
| TCGA-A2-A04V | BRCA | C1 | BRCA.LumA |
| TCGA-A2-A04X | BRCA | C2 | BRCA.Her2 |
| TCGA-A2-A0CX | BRCA | C2 | BRCA.Her2 |
| TCGA-A2-A0CZ | BRCA | C3 | BRCA.Normal |
| TCGA-A2-A0D2 | BRCA | C1 | BRCA.Basal |
| TCGA-A2-A0EW | BRCA | C3 | BRCA.LumA |
| TCGA-A2-A0T7 | BRCA | C1 | BRCA.LumA |
| TCGA-A2-A0YC | BRCA | C2 | BRCA.LumA |
| TCGA-A2-A0YF | BRCA | C4 | BRCA.LumA |
| TCGA-A2-A0YT | BRCA | C2 | BRCA.LumB |
| TCGA-A2-A1FX | BRCA | C4 | BRCA.LumB |
| TCGA-A2-A1G6 | BRCA | C3 | BRCA.Normal |
| TCGA-A2-A25C | BRCA | C2 | BRCA.LumB |
| TCGA-A2-A25D | BRCA | C2 | BRCA.LumA |
| TCGA-A2-A25E | BRCA | C1 | BRCA.LumB |
| TCGA-A2-A4RX | BRCA | C3 | BRCA.Basal |
| TCGA-A2-A4S2 | BRCA | C2 | BRCA.LumA |
| TCGA-A7-A0CG | BRCA | C3 | BRCA.LumA |
| TCGA-A7-A0D9 | BRCA | C1 | BRCA.Normal |
| TCGA-A7-A3IY | BRCA | C4 | BRCA.LumA |
| TCGA-A8-A06O | BRCA | C2 | BRCA.LumB |
| TCGA-A8-A06P | BRCA | C1 | BRCA.LumA |
| TCGA-A8-A06Z | BRCA | C4 | BRCA.LumB |
| TCGA-A8-A07G | BRCA | C1 | BRCA.LumA |
| TCGA-A8-A07L | BRCA | C1 | BRCA.LumB |
| TCGA-A8-A07Z | BRCA | C2 | BRCA.LumA |
| TCGA-A8-A082 | BRCA | C4 | BRCA.LumB |
| TCGA-A8-A08P | BRCA | C2 | BRCA.LumB |
| TCGA-A8-A096 | BRCA | C6 | BRCA.LumB |
| TCGA-A8-A09B | BRCA | C1 | BRCA.LumA |
| TCGA-AC-A3EH | BRCA | C1 | BRCA.LumA |
| TCGA-AC-A4ZE | BRCA | C3 | BRCA.LumA |
| TCGA-AC-A5EH | BRCA | C1 | BRCA.Her2 |
| TCGA-AN-A0AL | BRCA | C2 | BRCA.Basal |
| TCGA-AN-A0AT | BRCA | C1 | BRCA.Basal |
| TCGA-AN-A0FZ | BRCA | C1 | BRCA.LumA |
| TCGA-AN-A0XN | BRCA | C6 | BRCA.LumA |
| TCGA-AO-A0J4 | BRCA | C2 | BRCA.Basal |
| TCGA-AO-A0J5 | BRCA | C1 | BRCA.LumA |
| TCGA-AO-A0J9 | BRCA | C1 | BRCA.LumA |
| TCGA-AQ-A04L | BRCA | C1 | BRCA.LumA |
| TCGA-AR-A0TW | BRCA | C2 | BRCA.LumA |
| TCGA-AR-A1AJ | BRCA | C2 | BRCA.Basal |
| TCGA-AR-A1AL | BRCA | C3 | BRCA.LumA |
| TCGA-AR-A1AN | BRCA | C1 | BRCA.LumA |
| TCGA-AR-A1AO | BRCA | C2 | BRCA.Normal |
| TCGA-AR-A1AV | BRCA | C4 | BRCA.LumA |
| TCGA-AR-A24H | BRCA | C2 | BRCA.LumB |
| TCGA-AR-A24W | BRCA | C3 | BRCA.LumA |
| TCGA-AR-A2LK | BRCA | C1 | BRCA.LumB |
| TCGA-AR-A2LN | BRCA | C3 | BRCA.LumA |
| TCGA-AR-A5QQ | BRCA | C2 | BRCA.Basal |
| TCGA-B6-A0IQ | BRCA | C1 | BRCA.Basal |
| TCGA-B6-A0RH | BRCA | C2 | BRCA.Her2 |
| TCGA-B6-A0WW | BRCA | C2 | BRCA.LumB |
| TCGA-B6-A408 | BRCA | C2 | BRCA.LumA |
| TCGA-BH-A0BJ | BRCA | C3 | BRCA.Normal |
| TCGA-BH-A0BQ | BRCA | C2 | BRCA.Normal |
| TCGA-BH-A0DI | BRCA | C1 | BRCA.LumA |
| TCGA-BH-A0DS | BRCA | C3 | BRCA.LumA |
| TCGA-BH-A0DZ | BRCA | C1 | BRCA.Normal |
| TCGA-BH-A0E0 | BRCA | C1 | BRCA.Normal |
| TCGA-BH-A0E1 | BRCA | C1 | BRCA.Normal |
| TCGA-BH-A0E9 | BRCA | C3 | BRCA.LumA |
| TCGA-BH-A0H6 | BRCA | C1 | BRCA.LumA |
| TCGA-BH-A0HF | BRCA | C1 | BRCA.LumA |
| TCGA-BH-A1FB | BRCA | C3 | BRCA.Normal |
| TCGA-BH-A1FD | BRCA | C4 | BRCA.Normal |
| TCGA-BH-A1FG | BRCA | C2 | BRCA.Normal |
| TCGA-BH-A1FL | BRCA | C3 | BRCA.LumA |
| TCGA-BH-A201 | BRCA | C1 | BRCA.LumA |
| TCGA-BH-A208 | BRCA | C1 | BRCA.Normal |
| TCGA-BH-A28Q | BRCA | C3 | BRCA.LumA |
| TCGA-BH-A42V | BRCA | C3 | BRCA.LumA |
| TCGA-C8-A12L | BRCA | C1 | BRCA.Her2 |
| TCGA-C8-A12M | BRCA | C3 | BRCA.LumB |
| TCGA-C8-A12V | BRCA | C2 | BRCA.Basal |
| TCGA-C8-A134 | BRCA | C2 | BRCA.Basal |
| TCGA-C8-A26V | BRCA | C2 | BRCA.LumB |
| TCGA-C8-A273 | BRCA | C2 | BRCA.LumA |
| TCGA-C8-A27A | BRCA | C2 | BRCA.LumB |
| TCGA-C8-A8HP | BRCA | C1 | BRCA.Her2 |
| TCGA-D8-A143 | BRCA | C1 | BRCA.Basal |
| TCGA-D8-A1JF | BRCA | C3 | BRCA.Her2 |
| TCGA-D8-A1JN | BRCA | C3 | BRCA.LumA |
| TCGA-D8-A1X6 | BRCA | C4 | BRCA.LumB |
| TCGA-D8-A1X8 | BRCA | C3 | BRCA.LumA |
| TCGA-D8-A1XM | BRCA | C2 | BRCA.LumA |
| TCGA-D8-A1XO | BRCA | C3 | BRCA.LumA |
| TCGA-D8-A1Y2 | BRCA | C2 | BRCA.LumB |
| TCGA-D8-A27R | BRCA | C1 | BRCA.LumB |
| TCGA-E2-A108 | BRCA | C3 | BRCA.Normal |
| TCGA-E2-A14U | BRCA | C3 | BRCA.LumA |
| TCGA-E2-A14Y | BRCA | C2 | BRCA.Basal |
| TCGA-E2-A159 | BRCA | C2 | BRCA.Basal |
| TCGA-E2-A15C | BRCA | C3 | BRCA.LumA |
| TCGA-E2-A15O | BRCA | C1 | BRCA.LumA |
| TCGA-E2-A15S | BRCA | C1 | BRCA.LumB |
| TCGA-E2-A1IF | BRCA | C1 | BRCA.LumA |
| TCGA-E2-A1LA | BRCA | C2 | BRCA.LumA |
| TCGA-E2-A1LS | BRCA | C3 | BRCA.Normal |
| TCGA-E2-A573 | BRCA | C2 | BRCA.Basal |
| TCGA-E2-A9RU | BRCA | C2 | BRCA.LumB |
| TCGA-E9-A1N3 | BRCA | C4 | BRCA.LumA |
| TCGA-E9-A1NC | BRCA | C2 | BRCA.Basal |
| TCGA-E9-A1NG | BRCA | C6 | BRCA.Normal |
| TCGA-E9-A1R5 | BRCA | C1 | BRCA.LumA |
| TCGA-E9-A227 | BRCA | C1 | BRCA.LumA |
| TCGA-E9-A22A | BRCA | C2 | BRCA.LumB |
| TCGA-E9-A54Y | BRCA | C1 | BRCA.LumB |
| TCGA-EW-A1IX | BRCA | C3 | BRCA.LumA |
| TCGA-EW-A1P5 | BRCA | C2 | BRCA.LumA |
| TCGA-EW-A1P6 | BRCA | C1 | BRCA.LumA |
| TCGA-GM-A2DK | BRCA | C1 | BRCA.LumA |
| TCGA-GM-A3XN | BRCA | C1 | BRCA.LumA |
| TCGA-LD-A74U | BRCA | C6 | BRCA.LumA |
| TCGA-LL-A440 | BRCA | C6 | BRCA.LumA |
| TCGA-OL-A5DA | BRCA | C3 | BRCA.LumA |
| TCGA-OL-A66H | BRCA | C2 | BRCA.LumA |
| TCGA-OL-A66N | BRCA | C3 | BRCA.LumA |
| TCGA-OL-A66O | BRCA | C2 | BRCA.LumA |
| TCGA-S3-A6ZG | BRCA | C3 | BRCA.LumA |
| TCGA-W8-A86G | BRCA | C3 | BRCA.LumA |
| TCGA-XX-A899 | BRCA | C3 | BRCA.LumA |
| TCGA-3C-AAAU | BRCA | C1 | BRCA.LumA |
| TCGA-A1-A0SN | BRCA | C1 | BRCA.LumB |
| TCGA-A1-A0SO | BRCA | C1 | BRCA.Basal |
| TCGA-A2-A04Q | BRCA | C2 | BRCA.Basal |
| TCGA-A2-A04T | BRCA | C2 | BRCA.Basal |
| TCGA-A2-A0EM | BRCA | C1 | BRCA.LumA |
| TCGA-A2-A3XZ | BRCA | C2 | BRCA.Her2 |
| TCGA-A2-A4S0 | BRCA | C3 | BRCA.LumA |
| TCGA-A7-A26F | BRCA | C2 | BRCA.Basal |
| TCGA-A7-A3RF | BRCA | C4 | BRCA.LumA |
| TCGA-A7-A425 | BRCA | C3 | BRCA.LumA |
| TCGA-A7-A4SD | BRCA | C2 | BRCA.Basal |
| TCGA-A7-A4SF | BRCA | C1 | BRCA.Her2 |
| TCGA-A7-A56D | BRCA | C2 | BRCA.LumA |
| TCGA-A7-A6VW | BRCA | C2 | BRCA.Basal |
| TCGA-A8-A06U | BRCA | C2 | BRCA.LumA |
| TCGA-A8-A079 | BRCA | C1 | BRCA.LumB |
| TCGA-A8-A08T | BRCA | C1 | BRCA.LumA |
| TCGA-A8-A09G | BRCA | C2 | BRCA.Her2 |
| TCGA-A8-A0A4 | BRCA | C1 | BRCA.LumA |
| TCGA-A8-A0AB | BRCA | C1 | BRCA.LumA |
| TCGA-AC-A2BM | BRCA | C2 | BRCA.LumB |
| TCGA-AC-A3W6 | BRCA | C1 | BRCA.LumA |
| TCGA-AC-A5EI | BRCA | NA | NA |
| TCGA-AC-A62V | BRCA | C1 | BRCA.LumB |
| TCGA-AC-A6NO | BRCA | C1 | BRCA.LumA |
| TCGA-AN-A04A | BRCA | C1 | BRCA.LumA |
| TCGA-AN-A0AS | BRCA | C6 | BRCA.LumA |
| TCGA-AN-A0FJ | BRCA | C1 | BRCA.Basal |
| TCGA-AO-A03R | BRCA | C1 | BRCA.Normal |
| TCGA-AO-A0JD | BRCA | C2 | BRCA.LumB |
| TCGA-AO-A12H | BRCA | C4 | BRCA.LumA |
| TCGA-AQ-A1H3 | BRCA | C1 | BRCA.LumA |
| TCGA-AQ-A7U7 | BRCA | C2 | BRCA.LumA |
| TCGA-AR-A0U4 | BRCA | C4 | BRCA.Basal |
| TCGA-AR-A1AU | BRCA | C1 | BRCA.LumA |
| TCGA-AR-A251 | BRCA | C1 | BRCA.Basal |
| TCGA-AR-A2LM | BRCA | C3 | BRCA.LumA |
| TCGA-B6-A0WS | BRCA | C2 | BRCA.LumA |
| TCGA-B6-A0WX | BRCA | C1 | BRCA.Basal |
| TCGA-B6-A0X7 | BRCA | C2 | BRCA.LumA |
| TCGA-BH-A0AZ | BRCA | C1 | BRCA.Normal |
| TCGA-BH-A0B4 | BRCA | C1 | BRCA.LumA |
| TCGA-BH-A0B7 | BRCA | C1 | BRCA.Normal |
| TCGA-BH-A0BC | BRCA | C2 | BRCA.Normal |
| TCGA-BH-A0BL | BRCA | C1 | BRCA.Basal |
| TCGA-BH-A0BO | BRCA | C3 | BRCA.LumA |
| TCGA-BH-A0DG | BRCA | C2 | BRCA.Normal |
| TCGA-BH-A0DL | BRCA | C2 | BRCA.Normal |
| TCGA-BH-A0EI | BRCA | C1 | BRCA.LumA |
| TCGA-BH-A0RX | BRCA | C2 | BRCA.Basal |
| TCGA-BH-A0W3 | BRCA | C4 | BRCA.LumB |
| TCGA-BH-A0W7 | BRCA | C2 | BRCA.LumA |
| TCGA-BH-A18J | BRCA | C4 | BRCA.Normal |
| TCGA-BH-A18M | BRCA | C2 | BRCA.Normal |
| TCGA-BH-A18N | BRCA | C3 | BRCA.Normal |
| TCGA-BH-A1F8 | BRCA | C2 | BRCA.Normal |
| TCGA-BH-A1FM | BRCA | C4 | BRCA.Normal |
| TCGA-BH-A209 | BRCA | C2 | BRCA.Normal |
| TCGA-C8-A12N | BRCA | C2 | BRCA.LumA |
| TCGA-C8-A12P | BRCA | C2 | BRCA.Her2 |
| TCGA-C8-A138 | BRCA | C2 | BRCA.Her2 |
| TCGA-C8-A1HE | BRCA | C6 | BRCA.LumA |
| TCGA-C8-A1HO | BRCA | C2 | BRCA.LumA |
| TCGA-D8-A142 | BRCA | C1 | BRCA.Basal |
| TCGA-D8-A1JP | BRCA | C1 | BRCA.LumA |
| TCGA-D8-A1JS | BRCA | C4 | BRCA.LumA |
| TCGA-D8-A1XG | BRCA | C4 | BRCA.LumA |
| TCGA-D8-A1XV | BRCA | C4 | BRCA.LumA |
| TCGA-D8-A1Y3 | BRCA | C2 | BRCA.LumB |
| TCGA-D8-A27L | BRCA | C3 | BRCA.LumA |
| TCGA-D8-A27V | BRCA | C3 | BRCA.LumA |
| TCGA-D8-A27W | BRCA | C2 | BRCA.LumB |
| TCGA-E2-A107 | BRCA | C1 | BRCA.LumA |
| TCGA-E2-A14W | BRCA | C1 | BRCA.LumB |
| TCGA-E2-A150 | BRCA | C2 | BRCA.Basal |
| TCGA-E2-A154 | BRCA | C1 | BRCA.LumA |
| TCGA-E2-A156 | BRCA | C3 | BRCA.LumA |
| TCGA-E2-A1IH | BRCA | C1 | BRCA.LumA |
| TCGA-E2-A1IK | BRCA | C2 | BRCA.LumA |
| TCGA-E2-A1IN | BRCA | C1 | BRCA.LumA |
| TCGA-E2-A1L6 | BRCA | C1 | BRCA.LumA |
| TCGA-E2-A1LK | BRCA | C2 | BRCA.Basal |
| TCGA-E2-A1LL | BRCA | C1 | BRCA.Basal |
| TCGA-E9-A1N6 | BRCA | C4 | BRCA.Normal |
| TCGA-E9-A1QZ | BRCA | C2 | BRCA.LumA |
| TCGA-E9-A1RF | BRCA | C2 | BRCA.LumA |
| TCGA-E9-A249 | BRCA | C2 | BRCA.LumB |
| TCGA-E9-A54X | BRCA | C4 | BRCA.LumB |
| TCGA-EW-A1J6 | BRCA | C2 | BRCA.LumB |
| TCGA-EW-A1OW | BRCA | C2 | BRCA.Basal |
| TCGA-EW-A2FW | BRCA | C1 | BRCA.LumB |
| TCGA-GM-A2DM | BRCA | C3 | BRCA.LumA |
| TCGA-OK-A5Q2 | BRCA | C3 | BRCA.LumA |
| TCGA-OL-A5RX | BRCA | C3 | BRCA.LumA |
| TCGA-OL-A5RY | BRCA | C1 | BRCA.Normal |
| TCGA-OL-A6VQ | BRCA | C3 | BRCA.LumA |
| TCGA-S3-AA0Z | BRCA | C2 | BRCA.Basal |
| TCGA-WT-AB44 | BRCA | C1 | BRCA.LumA |
| TCGA-XX-A89A | BRCA | C3 | BRCA.LumA |
| TCGA-Z7-A8R6 | BRCA | C1 | BRCA.LumB |
| TCGA-A2-A04U | BRCA | C1 | BRCA.Basal |
| TCGA-A2-A0CK | BRCA | C1 | BRCA.LumA |
| TCGA-A2-A0CR | BRCA | C6 | BRCA.LumA |
| TCGA-A2-A0CT | BRCA | C1 | BRCA.LumA |
| TCGA-A2-A0CV | BRCA | C3 | BRCA.LumA |
| TCGA-A2-A0ER | BRCA | C1 | BRCA.LumA |
| TCGA-A2-A0YE | BRCA | C2 | BRCA.Basal |
| TCGA-A2-A1FV | BRCA | C3 | BRCA.LumA |
| TCGA-A2-A259 | BRCA | C1 | BRCA.LumA |
| TCGA-A2-A3KC | BRCA | C3 | BRCA.LumA |
| TCGA-A2-A3KD | BRCA | C3 | BRCA.LumA |
| TCGA-A2-A3XS | BRCA | C2 | BRCA.Basal |
| TCGA-A2-A3XU | BRCA | C1 | BRCA.Basal |
| TCGA-A7-A0CH | BRCA | C1 | BRCA.Normal |
| TCGA-A7-A13D | BRCA | C2 | BRCA.Basal |
| TCGA-A7-A13G | BRCA | C3 | BRCA.LumA |
| TCGA-A7-A26H | BRCA | C1 | BRCA.LumA |
| TCGA-A7-A4SA | BRCA | C2 | BRCA.LumA |
| TCGA-A7-A5ZV | BRCA | C4 | BRCA.Basal |
| TCGA-A7-A5ZW | BRCA | C1 | BRCA.LumA |
| TCGA-A8-A06R | BRCA | C2 | BRCA.LumB |
| TCGA-A8-A07J | BRCA | C6 | BRCA.LumA |
| TCGA-A8-A08C | BRCA | C3 | BRCA.LumA |
| TCGA-A8-A08H | BRCA | C6 | BRCA.Normal |
| TCGA-A8-A08I | BRCA | C2 | BRCA.LumB |
| TCGA-A8-A090 | BRCA | C2 | BRCA.LumA |
| TCGA-A8-A09C | BRCA | C1 | BRCA.LumB |
| TCGA-A8-A09D | BRCA | C6 | BRCA.LumA |
| TCGA-A8-A09Z | BRCA | C3 | BRCA.LumB |
| TCGA-A8-A0AD | BRCA | C4 | BRCA.LumA |
| TCGA-AC-A23H | BRCA | C1 | BRCA.Normal |
| TCGA-AC-A2FB | BRCA | C2 | BRCA.Normal |
| TCGA-AC-A2FG | BRCA | C3 | BRCA.LumA |
| TCGA-AC-A3W5 | BRCA | C1 | BRCA.Her2 |
| TCGA-AC-A3YI | BRCA | C1 | BRCA.Normal |
| TCGA-AN-A0FS | BRCA | C1 | BRCA.LumA |
| TCGA-AN-A0XP | BRCA | C2 | BRCA.LumA |
| TCGA-AO-A03V | BRCA | C1 | BRCA.LumA |
| TCGA-AO-A0JF | BRCA | C3 | BRCA.LumA |
| TCGA-AO-A124 | BRCA | C2 | BRCA.Basal |
| TCGA-AO-A12F | BRCA | C1 | BRCA.Basal |
| TCGA-AO-A1KP | BRCA | C1 | BRCA.LumB |
| TCGA-AQ-A0Y5 | BRCA | C4 | BRCA.LumA |
| TCGA-AR-A0TZ | BRCA | C1 | BRCA.LumA |
| TCGA-AR-A2LJ | BRCA | C6 | BRCA.LumA |
| TCGA-B6-A0IA | BRCA | C4 | BRCA.LumA |
| TCGA-B6-A0IG | BRCA | C2 | BRCA.LumA |
| TCGA-B6-A0IH | BRCA | C3 | BRCA.LumA |
| TCGA-B6-A0RE | BRCA | C1 | BRCA.Basal |
| TCGA-B6-A0RI | BRCA | C1 | BRCA.LumA |
| TCGA-B6-A0RS | BRCA | C1 | BRCA.Her2 |
| TCGA-B6-A40B | BRCA | C1 | BRCA.LumA |
| TCGA-BH-A0B5 | BRCA | C1 | BRCA.LumA |
| TCGA-BH-A0BP | BRCA | C1 | BRCA.LumA |
| TCGA-BH-A0C3 | BRCA | C2 | BRCA.Normal |
| TCGA-BH-A0DK | BRCA | C2 | BRCA.LumA |
| TCGA-BH-A0E6 | BRCA | C1 | BRCA.Basal |
| TCGA-BH-A0GZ | BRCA | C1 | BRCA.LumA |
| TCGA-BH-A0H3 | BRCA | C3 | BRCA.LumA |
| TCGA-BH-A0H9 | BRCA | C1 | BRCA.Normal |
| TCGA-BH-A0HP | BRCA | C1 | BRCA.LumA |
| TCGA-BH-A0HQ | BRCA | C1 | BRCA.LumA |
| TCGA-BH-A18F | BRCA | C1 | BRCA.LumA |
| TCGA-BH-A1EU | BRCA | C3 | BRCA.Normal |
| TCGA-BH-A1EX | BRCA | C6 | BRCA.LumA |
| TCGA-BH-A5IZ | BRCA | C1 | BRCA.Basal |
| TCGA-BH-A6R8 | BRCA | C2 | BRCA.LumA |
| TCGA-BH-A8FZ | BRCA | C2 | BRCA.Normal |
| TCGA-C8-A133 | BRCA | C4 | BRCA.LumA |
| TCGA-C8-A135 | BRCA | C1 | BRCA.Her2 |
| TCGA-C8-A1HK | BRCA | C2 | BRCA.Her2 |
| TCGA-C8-A1HL | BRCA | C1 | BRCA.LumB |
| TCGA-C8-A3M7 | BRCA | C3 | BRCA.LumA |
| TCGA-C8-A9FZ | BRCA | NA | NA |
| TCGA-D8-A1JA | BRCA | C4 | BRCA.Her2 |
| TCGA-D8-A1X7 | BRCA | C1 | BRCA.LumB |
| TCGA-D8-A27F | BRCA | C1 | BRCA.Basal |
| TCGA-D8-A27M | BRCA | C2 | BRCA.Basal |
| TCGA-D8-A27T | BRCA | C2 | BRCA.LumA |
| TCGA-E2-A106 | BRCA | C3 | BRCA.LumA |
| TCGA-E2-A10B | BRCA | C2 | BRCA.LumA |
| TCGA-E2-A10C | BRCA | C2 | BRCA.LumB |
| TCGA-E2-A15D | BRCA | C3 | BRCA.LumA |
| TCGA-E2-A15J | BRCA | C4 | BRCA.LumA |
| TCGA-E2-A15L | BRCA | C1 | BRCA.LumA |
| TCGA-E2-A15M | BRCA | C2 | BRCA.Normal |
| TCGA-E2-A1B0 | BRCA | C1 | BRCA.Her2 |
| TCGA-E2-A1B6 | BRCA | C2 | BRCA.Basal |
| TCGA-E2-A574 | BRCA | C1 | BRCA.Basal |
| TCGA-E9-A1N4 | BRCA | C6 | BRCA.Normal |
| TCGA-E9-A1RB | BRCA | C1 | BRCA.Normal |
| TCGA-E9-A228 | BRCA | C4 | BRCA.LumB |
| TCGA-E9-A22H | BRCA | C2 | BRCA.LumB |
| TCGA-E9-A5FK | BRCA | C2 | BRCA.LumA |
| TCGA-EW-A1J2 | BRCA | C3 | BRCA.LumA |
| TCGA-EW-A1OX | BRCA | C4 | BRCA.LumB |
| TCGA-EW-A1P1 | BRCA | C1 | BRCA.Normal |
| TCGA-EW-A1PB | BRCA | C2 | BRCA.Basal |
| TCGA-EW-A1PH | BRCA | C2 | BRCA.Basal |
| TCGA-GI-A2C8 | BRCA | C3 | BRCA.LumA |
| TCGA-GM-A2DD | BRCA | C2 | BRCA.Normal |
| TCGA-LL-A73Y | BRCA | C3 | BRCA.Basal |
| TCGA-LL-A7SZ | BRCA | C2 | BRCA.LumA |
| TCGA-PE-A5DC | BRCA | C2 | BRCA.LumA |
| TCGA-PL-A8LY | BRCA | C1 | BRCA.Normal |
| TCGA-PL-A8LZ | BRCA | C2 | BRCA.Basal |
| TCGA-S3-AA10 | BRCA | C2 | BRCA.Basal |
| TCGA-5L-AAT0 | BRCA | C3 | BRCA.LumA |
| TCGA-5T-A9QA | BRCA | C4 | BRCA.LumB |
| TCGA-A1-A0SI | BRCA | C2 | BRCA.LumB |
| TCGA-A1-A0SJ | BRCA | C1 | BRCA.LumA |
| TCGA-A1-A0SK | BRCA | C1 | BRCA.Basal |
| TCGA-A2-A04Y | BRCA | C2 | BRCA.LumB |
| TCGA-A2-A0CU | BRCA | C1 | BRCA.LumA |
| TCGA-A2-A0D3 | BRCA | C3 | BRCA.LumA |
| TCGA-A2-A0EV | BRCA | C2 | BRCA.LumA |
| TCGA-A2-A0EX | BRCA | C3 | BRCA.LumA |
| TCGA-A2-A0SW | BRCA | C2 | BRCA.LumB |
| TCGA-A2-A0T1 | BRCA | C1 | BRCA.Her2 |
| TCGA-A2-A0T4 | BRCA | C2 | BRCA.LumA |
| TCGA-A2-A0YD | BRCA | C1 | BRCA.LumA |
| TCGA-A2-A25F | BRCA | C2 | BRCA.Basal |
| TCGA-A2-A3XX | BRCA | C1 | BRCA.Basal |
| TCGA-A7-A0CD | BRCA | C4 | BRCA.LumA |
| TCGA-A7-A13F | BRCA | C4 | BRCA.Normal |
| TCGA-A7-A13H | BRCA | C3 | BRCA.LumA |
| TCGA-A7-A26G | BRCA | C1 | BRCA.Basal |
| TCGA-A7-A6VX | BRCA | C2 | BRCA.LumB |
| TCGA-A8-A08J | BRCA | C2 | BRCA.Her2 |
| TCGA-A8-A08L | BRCA | C1 | BRCA.Her2 |
| TCGA-A8-A094 | BRCA | C2 | BRCA.Her2 |
| TCGA-A8-A09I | BRCA | C2 | BRCA.LumB |
| TCGA-A8-A09Q | BRCA | C2 | BRCA.LumB |
| TCGA-A8-A09R | BRCA | C1 | BRCA.LumB |
| TCGA-A8-A0A6 | BRCA | C3 | BRCA.LumA |
| TCGA-A8-A0A9 | BRCA | C2 | BRCA.LumA |
| TCGA-AC-A2FO | BRCA | C3 | BRCA.LumA |
| TCGA-AC-A3QQ | BRCA | C1 | BRCA.LumA |
| TCGA-AC-A3TN | BRCA | C3 | BRCA.LumA |
| TCGA-AC-A62Y | BRCA | C4 | BRCA.LumA |
| TCGA-AC-A8OR | BRCA | C4 | BRCA.LumA |
| TCGA-AN-A04D | BRCA | C1 | BRCA.Basal |
| TCGA-AN-A0FV | BRCA | C4 | BRCA.Her2 |
| TCGA-AN-A0FX | BRCA | C1 | BRCA.Basal |
| TCGA-AN-A0FY | BRCA | C1 | BRCA.LumB |
| TCGA-AO-A03L | BRCA | C2 | BRCA.LumA |
| TCGA-AO-A0J2 | BRCA | C2 | BRCA.Her2 |
| TCGA-AO-A0JE | BRCA | C2 | BRCA.Her2 |
| TCGA-AO-A0JG | BRCA | C3 | BRCA.LumA |
| TCGA-AQ-A1H2 | BRCA | C4 | BRCA.LumA |
| TCGA-AR-A0TV | BRCA | C2 | BRCA.LumB |
| TCGA-AR-A24K | BRCA | C1 | BRCA.LumB |
| TCGA-AR-A24N | BRCA | C2 | BRCA.LumB |
| TCGA-AR-A24P | BRCA | C1 | BRCA.LumA |
| TCGA-AR-A24U | BRCA | C2 | BRCA.Her2 |
| TCGA-AR-A24V | BRCA | C2 | BRCA.LumA |
| TCGA-AR-A24X | BRCA | C1 | BRCA.LumA |
| TCGA-AR-A254 | BRCA | C6 | BRCA.Her2 |
| TCGA-AR-A2LQ | BRCA | C3 | BRCA.Normal |
| TCGA-B6-A0I1 | BRCA | C2 | BRCA.Basal |
| TCGA-B6-A0IC | BRCA | C3 | BRCA.LumA |
| TCGA-B6-A0IK | BRCA | C1 | BRCA.Her2 |
| TCGA-B6-A0RM | BRCA | C3 | BRCA.LumA |
| TCGA-B6-A0RN | BRCA | C3 | BRCA.LumA |
| TCGA-B6-A0RV | BRCA | C2 | BRCA.LumA |
| TCGA-B6-A0WV | BRCA | C1 | BRCA.LumB |
| TCGA-B6-A0X0 | BRCA | C3 | BRCA.LumA |
| TCGA-B6-A1KC | BRCA | C4 | BRCA.LumB |
| TCGA-B6-A1KI | BRCA | C3 | BRCA.LumA |
| TCGA-BH-A0BG | BRCA | C2 | BRCA.Basal |
| TCGA-BH-A0BW | BRCA | C2 | BRCA.Normal |
| TCGA-BH-A0DX | BRCA | C6 | BRCA.LumA |
| TCGA-BH-A0GY | BRCA | C2 | BRCA.LumA |
| TCGA-BH-A0H7 | BRCA | C2 | BRCA.Normal |
| TCGA-BH-A0HW | BRCA | C4 | BRCA.LumB |
| TCGA-BH-A0HX | BRCA | C1 | BRCA.LumA |
| TCGA-BH-A18H | BRCA | C2 | BRCA.LumA |
| TCGA-BH-A18P | BRCA | C3 | BRCA.Normal |
| TCGA-BH-A18R | BRCA | C1 | BRCA.Normal |
| TCGA-BH-A18T | BRCA | C1 | BRCA.Basal |
| TCGA-BH-A1ES | BRCA | C1 | BRCA.LumA |
| TCGA-BH-A28O | BRCA | C3 | BRCA.Normal |
| TCGA-BH-A5J0 | BRCA | C2 | BRCA.LumA |
| TCGA-C8-A12O | BRCA | C1 | BRCA.LumA |
| TCGA-C8-A12X | BRCA | C2 | BRCA.LumA |
| TCGA-C8-A1HJ | BRCA | C2 | BRCA.Basal |
| TCGA-C8-A26Y | BRCA | C1 | BRCA.Her2 |
| TCGA-C8-A275 | BRCA | C2 | BRCA.Her2 |
| TCGA-C8-A278 | BRCA | C2 | BRCA.Her2 |
| TCGA-D8-A1JU | BRCA | C3 | BRCA.LumA |
| TCGA-D8-A1XD | BRCA | C1 | BRCA.LumA |
| TCGA-D8-A1XR | BRCA | C3 | BRCA.LumB |
| TCGA-D8-A1XY | BRCA | C2 | BRCA.LumA |
| TCGA-D8-A4Z1 | BRCA | C3 | BRCA.LumA |
| TCGA-E2-A15G | BRCA | C3 | BRCA.LumA |
| TCGA-E2-A1BD | BRCA | C1 | BRCA.LumA |
| TCGA-E2-A1LB | BRCA | C1 | BRCA.Normal |
| TCGA-E2-A1LE | BRCA | C1 | BRCA.Her2 |
| TCGA-E2-A570 | BRCA | C1 | BRCA.LumA |
| TCGA-E9-A1NA | BRCA | C4 | NA |
| TCGA-E9-A1R4 | BRCA | C2 | BRCA.LumB |
| TCGA-E9-A1R7 | BRCA | C3 | BRCA.Normal |
| TCGA-E9-A1RH | BRCA | C1 | BRCA.Normal |
| TCGA-E9-A22G | BRCA | C2 | BRCA.Basal |
| TCGA-E9-A247 | BRCA | C2 | BRCA.LumA |
| TCGA-E9-A24A | BRCA | C4 | BRCA.LumA |
| TCGA-E9-A3X8 | BRCA | C1 | BRCA.LumA |
| TCGA-E9-A5UO | BRCA | C4 | BRCA.LumB |
| TCGA-EW-A1PD | BRCA | C1 | BRCA.LumA |
| TCGA-EW-A6SB | BRCA | C2 | BRCA.Basal |
| TCGA-EW-A6SD | BRCA | C1 | BRCA.Her2 |
| TCGA-GM-A2DO | BRCA | C2 | BRCA.LumB |
| TCGA-GM-A3NY | BRCA | C1 | BRCA.LumA |
| TCGA-HN-A2NL | BRCA | C2 | BRCA.Basal |
| TCGA-JL-A3YX | BRCA | C1 | BRCA.LumA |
| TCGA-LL-A442 | BRCA | C4 | BRCA.LumA |
| TCGA-LL-A6FR | BRCA | C2 | BRCA.Basal |
| TCGA-LQ-A4E4 | BRCA | C1 | BRCA.LumA |
| TCGA-S3-A6ZH | BRCA | C1 | BRCA.LumA |
| TCGA-S3-AA11 | BRCA | C4 | BRCA.LumA |
| TCGA-A1-A0SB | BRCA | C1 | BRCA.Normal |
| TCGA-A1-A0SF | BRCA | C1 | BRCA.LumA |
| TCGA-A2-A0CS | BRCA | C1 | BRCA.LumA |
| TCGA-A2-A0D0 | BRCA | C2 | BRCA.Basal |
| TCGA-A2-A0EN | BRCA | C3 | BRCA.LumA |
| TCGA-A2-A0EP | BRCA | C3 | BRCA.LumA |
| TCGA-A2-A0EQ | BRCA | C2 | BRCA.Her2 |
| TCGA-A2-A0T0 | BRCA | C1 | BRCA.Basal |
| TCGA-A2-A0YH | BRCA | C2 | BRCA.LumB |
| TCGA-A2-A1G1 | BRCA | C2 | BRCA.Basal |
| TCGA-A2-A3XV | BRCA | C1 | BRCA.Her2 |
| TCGA-A2-A3XW | BRCA | C2 | BRCA.Normal |
| TCGA-A2-A3XY | BRCA | C2 | BRCA.Basal |
| TCGA-A2-A4RW | BRCA | C3 | BRCA.LumA |
| TCGA-A7-A0CE | BRCA | C2 | BRCA.Normal |
| TCGA-A7-A426 | BRCA | C3 | BRCA.LumA |
| TCGA-A8-A075 | BRCA | C1 | BRCA.LumB |
| TCGA-A8-A08G | BRCA | C2 | BRCA.LumB |
| TCGA-A8-A08R | BRCA | C2 | BRCA.Basal |
| TCGA-A8-A092 | BRCA | C2 | BRCA.LumB |
| TCGA-A8-A097 | BRCA | C1 | BRCA.LumA |
| TCGA-A8-A09N | BRCA | C1 | BRCA.LumB |
| TCGA-AC-A23E | BRCA | C4 | BRCA.LumA |
| TCGA-AC-A3YJ | BRCA | C3 | BRCA.LumA |
| TCGA-AC-A6IX | BRCA | C1 | BRCA.LumA |
| TCGA-AC-A8OP | BRCA | C3 | BRCA.LumA |
| TCGA-AC-A8OQ | BRCA | C1 | BRCA.Basal |
| TCGA-AN-A049 | BRCA | C2 | BRCA.LumA |
| TCGA-AN-A0XR | BRCA | C2 | BRCA.LumB |
| TCGA-AO-A03M | BRCA | C2 | BRCA.LumA |
| TCGA-AO-A0JL | BRCA | C1 | BRCA.Basal |
| TCGA-AO-A129 | BRCA | C2 | BRCA.Basal |
| TCGA-AO-A12A | BRCA | C1 | BRCA.LumA |
| TCGA-AO-A12B | BRCA | C4 | BRCA.LumA |
| TCGA-AO-A12D | BRCA | C2 | BRCA.Her2 |
| TCGA-AO-A12G | BRCA | C1 | BRCA.LumA |
| TCGA-AQ-A04H | BRCA | C1 | BRCA.LumB |
| TCGA-AR-A0TS | BRCA | C2 | BRCA.Basal |
| TCGA-AR-A0TT | BRCA | C6 | BRCA.LumB |
| TCGA-AR-A0U0 | BRCA | C2 | BRCA.Basal |
| TCGA-AR-A1AS | BRCA | C4 | BRCA.LumA |
| TCGA-AR-A1AW | BRCA | C2 | BRCA.LumB |
| TCGA-AR-A24O | BRCA | C2 | BRCA.LumA |
| TCGA-AR-A24T | BRCA | C6 | BRCA.LumA |
| TCGA-AR-A2LH | BRCA | C2 | BRCA.Normal |
| TCGA-AR-A5QN | BRCA | C2 | BRCA.LumA |
| TCGA-B6-A0IE | BRCA | C1 | BRCA.LumA |
| TCGA-B6-A0RQ | BRCA | C3 | BRCA.Normal |
| TCGA-B6-A0WY | BRCA | C2 | BRCA.LumA |
| TCGA-B6-A0X5 | BRCA | C4 | BRCA.LumB |
| TCGA-B6-A1KF | BRCA | C1 | BRCA.Basal |
| TCGA-B6-A40C | BRCA | C1 | BRCA.LumA |
| TCGA-BH-A0AV | BRCA | C1 | BRCA.Basal |
| TCGA-BH-A0AY | BRCA | C1 | BRCA.Normal |
| TCGA-BH-A0EA | BRCA | C3 | BRCA.LumA |
| TCGA-BH-A0EB | BRCA | C6 | BRCA.LumA |
| TCGA-BH-A18I | BRCA | C1 | BRCA.LumA |
| TCGA-BH-A1FJ | BRCA | C2 | BRCA.Normal |
| TCGA-C8-A12K | BRCA | C2 | BRCA.Basal |
| TCGA-C8-A26X | BRCA | C2 | BRCA.Her2 |
| TCGA-C8-A26Z | BRCA | C1 | BRCA.LumA |
| TCGA-C8-A274 | BRCA | C4 | BRCA.LumB |
| TCGA-D8-A13Y | BRCA | C4 | BRCA.LumB |
| TCGA-D8-A13Z | BRCA | C2 | BRCA.Her2 |
| TCGA-D8-A140 | BRCA | C2 | BRCA.LumA |
| TCGA-D8-A147 | BRCA | C2 | BRCA.Basal |
| TCGA-D8-A1JH | BRCA | C3 | BRCA.LumA |
| TCGA-D8-A1XF | BRCA | C6 | BRCA.LumB |
| TCGA-D8-A1XL | BRCA | C2 | BRCA.LumB |
| TCGA-D8-A1XU | BRCA | C6 | BRCA.LumA |
| TCGA-D8-A27G | BRCA | C1 | BRCA.LumA |
| TCGA-E2-A14N | BRCA | C2 | BRCA.Basal |
| TCGA-E2-A14Z | BRCA | C2 | BRCA.LumA |
| TCGA-E2-A155 | BRCA | C1 | BRCA.LumB |
| TCGA-E2-A15T | BRCA | C3 | BRCA.LumB |
| TCGA-E2-A1B1 | BRCA | C2 | BRCA.LumA |
| TCGA-E2-A1LG | BRCA | C2 | BRCA.Basal |
| TCGA-E2-A2P5 | BRCA | C1 | BRCA.LumA |
| TCGA-E2-A56Z | BRCA | C2 | BRCA.LumB |
| TCGA-E9-A1N5 | BRCA | C1 | BRCA.Normal |
| TCGA-E9-A1N8 | BRCA | C1 | BRCA.Basal |
| TCGA-E9-A1R6 | BRCA | C1 | BRCA.LumA |
| TCGA-E9-A1RE | BRCA | C1 | BRCA.LumB |
| TCGA-E9-A22B | BRCA | C2 | BRCA.LumA |
| TCGA-E9-A22D | BRCA | C2 | BRCA.LumB |
| TCGA-E9-A22E | BRCA | C2 | BRCA.LumB |
| TCGA-E9-A244 | BRCA | C1 | BRCA.Basal |
| TCGA-E9-A295 | BRCA | C1 | BRCA.LumA |
| TCGA-E9-A2JT | BRCA | C6 | BRCA.LumA |
| TCGA-E9-A3HO | BRCA | C2 | BRCA.LumB |
| TCGA-EW-A1IW | BRCA | C1 | BRCA.LumA |
| TCGA-EW-A1IY | BRCA | C2 | BRCA.LumB |
| TCGA-EW-A1J1 | BRCA | C2 | BRCA.LumA |
| TCGA-EW-A3E8 | BRCA | C2 | BRCA.LumA |
| TCGA-EW-A3U0 | BRCA | C2 | BRCA.Basal |
| TCGA-EW-A6SC | BRCA | C1 | BRCA.LumA |
| TCGA-GM-A2DB | BRCA | C2 | BRCA.Her2 |
| TCGA-GM-A2DF | BRCA | C2 | BRCA.Basal |
| TCGA-GM-A3XG | BRCA | C1 | BRCA.LumA |
| TCGA-LL-A441 | BRCA | C2 | BRCA.Normal |
| TCGA-LL-A6FP | BRCA | C4 | BRCA.LumA |
| TCGA-LL-A7T0 | BRCA | C2 | BRCA.LumB |
| TCGA-LL-A8F5 | BRCA | C2 | BRCA.Basal |
| TCGA-OL-A5D6 | BRCA | C1 | BRCA.Normal |
| TCGA-OL-A5D7 | BRCA | C2 | BRCA.Basal |
| TCGA-OL-A5RV | BRCA | C3 | BRCA.LumA |
| TCGA-OL-A66J | BRCA | C1 | BRCA.LumA |
| TCGA-OL-A66K | BRCA | C3 | BRCA.LumA |
| TCGA-OL-A97C | BRCA | C1 | BRCA.Normal |
| TCGA-S3-AA12 | BRCA | C3 | BRCA.LumA |
| TCGA-UU-A93S | BRCA | C2 | BRCA.Her2 |
| TCGA-A2-A04P | BRCA | C2 | BRCA.Basal |
| TCGA-A2-A0CY | BRCA | C1 | BRCA.LumB |
| TCGA-A2-A0ET | BRCA | C4 | BRCA.LumA |
| TCGA-A2-A0EY | BRCA | C1 | BRCA.LumB |
| TCGA-A2-A0ST | BRCA | C2 | BRCA.Basal |
| TCGA-A2-A0SV | BRCA | C2 | BRCA.LumB |
| TCGA-A2-A0T3 | BRCA | C1 | BRCA.LumB |
| TCGA-A2-A1FZ | BRCA | C3 | BRCA.LumA |
| TCGA-A2-A1G0 | BRCA | C3 | BRCA.LumA |
| TCGA-A2-A25A | BRCA | C1 | BRCA.Normal |
| TCGA-A2-A3Y0 | BRCA | C2 | BRCA.Basal |
| TCGA-A7-A0DB | BRCA | C6 | BRCA.Normal |
| TCGA-A7-A13E | BRCA | C1 | BRCA.Normal |
| TCGA-A7-A26E | BRCA | C3 | BRCA.LumA |
| TCGA-A7-A3J0 | BRCA | C3 | BRCA.LumA |
| TCGA-A7-A4SC | BRCA | C3 | BRCA.LumA |
| TCGA-A7-A5ZX | BRCA | C3 | BRCA.LumA |
| TCGA-A7-A6VV | BRCA | C1 | BRCA.Basal |
| TCGA-A8-A06Q | BRCA | C4 | BRCA.LumB |
| TCGA-A8-A06T | BRCA | C2 | BRCA.LumA |
| TCGA-A8-A076 | BRCA | C2 | BRCA.LumB |
| TCGA-A8-A07E | BRCA | C1 | BRCA.LumA |
| TCGA-A8-A07F | BRCA | C3 | BRCA.LumA |
| TCGA-A8-A07R | BRCA | C2 | BRCA.Basal |
| TCGA-A8-A07S | BRCA | C4 | BRCA.LumB |
| TCGA-A8-A07W | BRCA | C1 | BRCA.LumB |
| TCGA-A8-A086 | BRCA | C1 | BRCA.LumA |
| TCGA-A8-A08X | BRCA | C1 | BRCA.Her2 |
| TCGA-A8-A08Z | BRCA | C6 | BRCA.LumA |
| TCGA-A8-A091 | BRCA | C3 | BRCA.LumA |
| TCGA-A8-A09A | BRCA | C3 | BRCA.LumA |
| TCGA-A8-A09W | BRCA | C2 | BRCA.LumB |
| TCGA-AC-A2BK | BRCA | C1 | BRCA.Basal |
| TCGA-AC-A2FE | BRCA | C6 | BRCA.LumA |
| TCGA-AC-A2FF | BRCA | C3 | BRCA.Normal |
| TCGA-AC-A3OD | BRCA | C2 | BRCA.LumA |
| TCGA-AC-A3TM | BRCA | C1 | BRCA.LumA |
| TCGA-AC-A5XS | BRCA | C2 | BRCA.LumA |
| TCGA-AC-A5XU | BRCA | C1 | BRCA.LumA |
| TCGA-AC-A6IV | BRCA | C6 | BRCA.LumA |
| TCGA-AC-A6IW | BRCA | C2 | BRCA.Basal |
| TCGA-AC-A7VC | BRCA | C4 | BRCA.Basal |
| TCGA-AN-A03X | BRCA | C1 | BRCA.LumA |
| TCGA-AN-A0AJ | BRCA | C2 | BRCA.LumB |
| TCGA-AN-A0FF | BRCA | C2 | BRCA.LumB |
| TCGA-AN-A0XL | BRCA | C1 | BRCA.LumA |
| TCGA-AN-A0XU | BRCA | C1 | BRCA.Basal |
| TCGA-AO-A0J7 | BRCA | C1 | BRCA.LumB |
| TCGA-AO-A0JB | BRCA | C1 | BRCA.Normal |
| TCGA-AO-A0JC | BRCA | C2 | BRCA.LumA |
| TCGA-AO-A12E | BRCA | C4 | BRCA.LumA |
| TCGA-AO-A1KQ | BRCA | C2 | BRCA.LumB |
| TCGA-AQ-A54N | BRCA | C4 | BRCA.Basal |
| TCGA-AR-A0TX | BRCA | C3 | BRCA.Her2 |
| TCGA-AR-A0TY | BRCA | C1 | BRCA.LumB |
| TCGA-AR-A0U1 | BRCA | NA | BRCA.Basal |
| TCGA-AR-A0U2 | BRCA | C3 | BRCA.LumB |
| TCGA-AR-A0U3 | BRCA | C1 | BRCA.LumA |
| TCGA-AR-A1AQ | BRCA | C2 | BRCA.Basal |
| TCGA-AR-A1AT | BRCA | C2 | BRCA.Her2 |
| TCGA-AR-A255 | BRCA | C2 | BRCA.LumA |
| TCGA-AR-A5QP | BRCA | C1 | BRCA.LumA |
| TCGA-B6-A3ZX | BRCA | C2 | BRCA.Basal |
| TCGA-BH-A0AW | BRCA | C2 | BRCA.Her2 |
| TCGA-BH-A0B3 | BRCA | C2 | BRCA.Normal |
| TCGA-BH-A0BA | BRCA | C3 | BRCA.Normal |
| TCGA-BH-A0BM | BRCA | C3 | BRCA.Normal |
| TCGA-BH-A0BR | BRCA | C1 | BRCA.LumA |
| TCGA-BH-A0BZ | BRCA | C2 | BRCA.Normal |
| TCGA-BH-A0C7 | BRCA | C2 | BRCA.LumB |
| TCGA-BH-A0H0 | BRCA | C1 | BRCA.LumB |
| TCGA-BH-A0W5 | BRCA | C3 | BRCA.LumA |
| TCGA-BH-A18L | BRCA | C4 | BRCA.Normal |
| TCGA-BH-A18S | BRCA | C3 | BRCA.Normal |
| TCGA-BH-A1ET | BRCA | C3 | BRCA.Normal |
| TCGA-BH-A1FN | BRCA | C2 | BRCA.Normal |
| TCGA-BH-A1FR | BRCA | C3 | BRCA.Normal |
| TCGA-BH-A202 | BRCA | C1 | BRCA.LumB |
| TCGA-BH-A204 | BRCA | C4 | BRCA.Normal |
| TCGA-BH-A8G0 | BRCA | C3 | BRCA.LumA |
| TCGA-BH-AB28 | BRCA | C3 | BRCA.LumA |
| TCGA-C8-A12Z | BRCA | C1 | BRCA.Her2 |
| TCGA-C8-A1HM | BRCA | C2 | BRCA.LumB |
| TCGA-C8-A8HQ | BRCA | C1 | BRCA.LumB |
| TCGA-D8-A145 | BRCA | C3 | BRCA.LumA |
| TCGA-D8-A1J8 | BRCA | C2 | BRCA.LumB |
| TCGA-D8-A1JE | BRCA | C4 | BRCA.LumB |
| TCGA-D8-A1JG | BRCA | C2 | BRCA.Her2 |
| TCGA-D8-A1X9 | BRCA | C2 | BRCA.LumA |
| TCGA-D8-A1XA | BRCA | C2 | BRCA.LumA |
| TCGA-D8-A1XJ | BRCA | C1 | BRCA.Her2 |
| TCGA-D8-A1XQ | BRCA | C2 | BRCA.Basal |
| TCGA-D8-A27E | BRCA | C3 | BRCA.LumA |
| TCGA-D8-A27H | BRCA | C1 | BRCA.Basal |
| TCGA-D8-A27K | BRCA | C3 | BRCA.LumA |
| TCGA-D8-A27P | BRCA | C3 | BRCA.LumA |
| TCGA-D8-A73W | BRCA | C2 | BRCA.LumA |
| TCGA-E2-A105 | BRCA | C1 | BRCA.LumA |
| TCGA-E2-A109 | BRCA | C2 | BRCA.LumB |
| TCGA-E2-A10E | BRCA | C2 | BRCA.LumA |
| TCGA-E2-A14P | BRCA | C1 | BRCA.Her2 |
| TCGA-E2-A14R | BRCA | C2 | BRCA.Basal |
| TCGA-E2-A14V | BRCA | C1 | BRCA.Her2 |
| TCGA-E2-A152 | BRCA | C1 | BRCA.Her2 |
| TCGA-E2-A158 | BRCA | C1 | BRCA.Normal |
| TCGA-E2-A15E | BRCA | C1 | BRCA.LumA |
| TCGA-E2-A15K | BRCA | C1 | BRCA.Normal |
| TCGA-E2-A1BC | BRCA | C3 | BRCA.Normal |
| TCGA-E2-A1L9 | BRCA | C2 | BRCA.LumA |
| TCGA-E2-A2P6 | BRCA | C3 | BRCA.LumA |
| TCGA-E9-A1NI | BRCA | C2 | BRCA.LumB |
| TCGA-E9-A1RD | BRCA | C4 | BRCA.Normal |
| TCGA-E9-A226 | BRCA | C2 | BRCA.LumB |
| TCGA-E9-A243 | BRCA | C2 | BRCA.Basal |
| TCGA-E9-A245 | BRCA | C1 | BRCA.LumA |
| TCGA-E9-A3Q9 | BRCA | C3 | BRCA.LumA |
| TCGA-EW-A1OZ | BRCA | C1 | BRCA.LumB |
| TCGA-EW-A1P4 | BRCA | C1 | BRCA.Basal |
| TCGA-EW-A2FV | BRCA | C2 | BRCA.LumA |
| TCGA-GI-A2C9 | BRCA | C2 | BRCA.Normal |
| TCGA-GM-A3XL | BRCA | C2 | BRCA.Basal |
| TCGA-GM-A4E0 | BRCA | C3 | BRCA.LumA |
| TCGA-LD-A7W5 | BRCA | C2 | BRCA.LumA |
| TCGA-LL-A5YN | BRCA | C2 | BRCA.LumA |
| TCGA-OL-A5RW | BRCA | C2 | BRCA.Basal |
| TCGA-OL-A66P | BRCA | C2 | BRCA.Her2 |
| TCGA-OL-A6VR | BRCA | C4 | BRCA.LumA |
| TCGA-PE-A5DE | BRCA | C2 | BRCA.LumA |
| TCGA-PL-A8LX | BRCA | C4 | BRCA.LumA |
| TCGA-S3-A6ZF | BRCA | C2 | BRCA.LumB |
| TCGA-S3-AA17 | BRCA | C2 | BRCA.LumB |
| TCGA-3C-AALJ | BRCA | C1 | BRCA.LumB |
| TCGA-A2-A0EO | BRCA | C3 | BRCA.LumA |
| TCGA-A2-A0YG | BRCA | C1 | BRCA.LumB |
| TCGA-A2-A0YJ | BRCA | C1 | BRCA.Basal |
| TCGA-A2-A3XT | BRCA | C2 | BRCA.Basal |
| TCGA-A2-A4S3 | BRCA | C2 | BRCA.LumB |
| TCGA-A7-A26I | BRCA | C4 | BRCA.Basal |
| TCGA-A7-A26J | BRCA | C2 | BRCA.LumA |
| TCGA-A7-A3J1 | BRCA | C2 | BRCA.LumA |
| TCGA-A8-A085 | BRCA | C4 | BRCA.LumB |
| TCGA-A8-A095 | BRCA | C1 | BRCA.LumB |
| TCGA-A8-A09M | BRCA | C2 | BRCA.LumB |
| TCGA-A8-A0A7 | BRCA | C2 | BRCA.Her2 |
| TCGA-AC-A3QP | BRCA | C1 | BRCA.LumA |
| TCGA-AN-A03Y | BRCA | C1 | BRCA.LumB |
| TCGA-AN-A046 | BRCA | C2 | BRCA.LumA |
| TCGA-AN-A0FD | BRCA | C2 | BRCA.LumA |
| TCGA-AN-A0FN | BRCA | C6 | BRCA.LumA |
| TCGA-AN-A0XS | BRCA | C3 | BRCA.LumA |
| TCGA-AN-A0XT | BRCA | C3 | BRCA.LumA |
| TCGA-AN-A0XW | BRCA | C1 | BRCA.LumA |
| TCGA-AO-A03P | BRCA | C2 | BRCA.LumB |
| TCGA-AO-A0J6 | BRCA | C2 | BRCA.Basal |
| TCGA-AO-A0JI | BRCA | C4 | BRCA.LumA |
| TCGA-AO-A0JJ | BRCA | C6 | BRCA.LumA |
| TCGA-AO-A1KO | BRCA | C3 | BRCA.Normal |
| TCGA-AO-A1KR | BRCA | C2 | BRCA.Basal |
| TCGA-AR-A0TP | BRCA | C1 | BRCA.Basal |
| TCGA-AR-A0TR | BRCA | C3 | BRCA.LumA |
| TCGA-AR-A1AH | BRCA | C1 | BRCA.Basal |
| TCGA-AR-A1AR | BRCA | C2 | BRCA.Basal |
| TCGA-AR-A1AX | BRCA | C2 | BRCA.LumA |
| TCGA-AR-A24L | BRCA | C2 | BRCA.LumA |
| TCGA-AR-A24S | BRCA | C2 | BRCA.LumB |
| TCGA-AR-A256 | BRCA | C2 | BRCA.Basal |
| TCGA-B6-A0IN | BRCA | C1 | BRCA.LumB |
| TCGA-B6-A0IO | BRCA | C1 | BRCA.LumA |
| TCGA-B6-A0RG | BRCA | C2 | BRCA.LumA |
| TCGA-B6-A0RO | BRCA | C2 | BRCA.LumA |
| TCGA-B6-A0X4 | BRCA | C4 | BRCA.LumA |
| TCGA-B6-A1KN | BRCA | C2 | BRCA.LumB |
| TCGA-B6-A400 | BRCA | C2 | BRCA.Basal |
| TCGA-BH-A0AU | BRCA | C2 | BRCA.Normal |
| TCGA-BH-A0B0 | BRCA | C2 | BRCA.LumA |
| TCGA-BH-A0BF | BRCA | C2 | BRCA.LumB |
| TCGA-BH-A0BV | BRCA | C2 | BRCA.Normal |
| TCGA-BH-A0C1 | BRCA | C1 | BRCA.LumA |
| TCGA-BH-A0DO | BRCA | C3 | BRCA.Normal |
| TCGA-BH-A0DQ | BRCA | C2 | BRCA.LumA |
| TCGA-BH-A0E2 | BRCA | C1 | BRCA.LumA |
| TCGA-BH-A0HA | BRCA | C2 | BRCA.Normal |
| TCGA-BH-A0HI | BRCA | C1 | BRCA.LumA |
| TCGA-BH-A0HK | BRCA | C1 | BRCA.Normal |
| TCGA-BH-A0HL | BRCA | C4 | BRCA.LumA |
| TCGA-BH-A0HN | BRCA | C4 | BRCA.LumA |
| TCGA-BH-A0W4 | BRCA | C1 | BRCA.LumA |
| TCGA-BH-A18G | BRCA | C1 | BRCA.Basal |
| TCGA-BH-A18V | BRCA | C2 | BRCA.Normal |
| TCGA-BH-A1EY | BRCA | C3 | BRCA.LumA |
| TCGA-BH-A1F0 | BRCA | C2 | BRCA.Normal |
| TCGA-C8-A12U | BRCA | C2 | BRCA.LumB |
| TCGA-C8-A12Y | BRCA | C2 | BRCA.LumA |
| TCGA-D8-A1JB | BRCA | C2 | BRCA.LumA |
| TCGA-D8-A1XT | BRCA | C2 | BRCA.Her2 |
| TCGA-D8-A3Z6 | BRCA | C1 | BRCA.LumA |
| TCGA-E2-A10A | BRCA | C4 | BRCA.LumA |
| TCGA-E2-A14O | BRCA | C1 | BRCA.LumB |
| TCGA-E2-A14X | BRCA | C1 | BRCA.Basal |
| TCGA-E2-A153 | BRCA | C6 | BRCA.Normal |
| TCGA-E2-A15P | BRCA | C2 | BRCA.LumA |
| TCGA-E2-A1AZ | BRCA | C2 | BRCA.Basal |
| TCGA-E2-A1B4 | BRCA | C3 | BRCA.LumA |
| TCGA-E2-A1B5 | BRCA | C2 | BRCA.LumA |
| TCGA-E2-A1IG | BRCA | C1 | BRCA.Normal |
| TCGA-E2-A1II | BRCA | C2 | BRCA.Basal |
| TCGA-E2-A1IU | BRCA | C3 | BRCA.LumA |
| TCGA-E2-A576 | BRCA | C1 | BRCA.LumA |
| TCGA-E9-A1ND | BRCA | C2 | BRCA.Normal |
| TCGA-E9-A1R2 | BRCA | C2 | BRCA.LumA |
| TCGA-E9-A2JS | BRCA | C2 | BRCA.LumB |
| TCGA-EW-A1IZ | BRCA | C2 | BRCA.LumA |
| TCGA-EW-A1J3 | BRCA | C1 | BRCA.LumA |
| TCGA-EW-A1P7 | BRCA | C3 | BRCA.Normal |
| TCGA-EW-A1PF | BRCA | C1 | BRCA.LumA |
| TCGA-GM-A3NW | BRCA | C1 | BRCA.LumA |
| TCGA-JL-A3YW | BRCA | C1 | BRCA.Her2 |
| TCGA-LL-A5YM | BRCA | C1 | BRCA.LumB |
| TCGA-OL-A66L | BRCA | C1 | BRCA.LumA |
| TCGA-UL-AAZ6 | BRCA | C2 | BRCA.LumA |
| TCGA-V7-A7HQ | BRCA | C1 | BRCA.LumA |
| TCGA-3C-AALI | BRCA | C1 | BRCA.Her2 |
| TCGA-A1-A0SE | BRCA | C1 | BRCA.LumA |
| TCGA-A1-A0SG | BRCA | C3 | BRCA.LumA |
| TCGA-A1-A0SH | BRCA | C6 | BRCA.LumA |
| TCGA-A1-A0SP | BRCA | C2 | BRCA.Basal |
| TCGA-A1-A0SQ | BRCA | C3 | BRCA.LumA |
| TCGA-A2-A04N | BRCA | C1 | BRCA.LumA |
| TCGA-A2-A0CL | BRCA | C2 | BRCA.Normal |
| TCGA-A2-A0CM | BRCA | C2 | BRCA.Basal |
| TCGA-A2-A0CP | BRCA | C1 | BRCA.LumA |
| TCGA-A2-A0D1 | BRCA | C1 | BRCA.Her2 |
| TCGA-A2-A0D4 | BRCA | C1 | BRCA.LumB |
| TCGA-A2-A0SU | BRCA | C1 | BRCA.LumA |
| TCGA-A2-A0T6 | BRCA | C3 | BRCA.LumA |
| TCGA-A2-A0YI | BRCA | C3 | BRCA.LumA |
| TCGA-A2-A0YL | BRCA | C1 | BRCA.LumA |
| TCGA-A2-A0YM | BRCA | C2 | BRCA.Basal |
| TCGA-A2-A25B | BRCA | C2 | BRCA.LumB |
| TCGA-A2-A4RY | BRCA | C3 | BRCA.Normal |
| TCGA-A7-A0CJ | BRCA | C2 | BRCA.LumB |
| TCGA-A7-A0DA | BRCA | C1 | BRCA.Basal |
| TCGA-A7-A0DC | BRCA | NA | BRCA.Normal |
| TCGA-A7-A4SB | BRCA | C3 | BRCA.LumA |
| TCGA-A8-A06X | BRCA | C2 | BRCA.LumB |
| TCGA-A8-A07P | BRCA | C1 | BRCA.LumA |
| TCGA-A8-A093 | BRCA | C1 | BRCA.LumA |
| TCGA-A8-A09T | BRCA | C4 | BRCA.LumA |
| TCGA-A8-A09V | BRCA | C3 | BRCA.LumA |
| TCGA-A8-A0A1 | BRCA | C3 | BRCA.LumA |
| TCGA-AC-A23C | BRCA | C2 | BRCA.LumA |
| TCGA-AC-A2FM | BRCA | C1 | BRCA.Normal |
| TCGA-AC-A2QJ | BRCA | C1 | BRCA.Basal |
| TCGA-AC-A3BB | BRCA | C3 | BRCA.LumA |
| TCGA-AC-A3HN | BRCA | C3 | BRCA.LumA |
| TCGA-AN-A041 | BRCA | C1 | BRCA.LumA |
| TCGA-AN-A0FT | BRCA | C1 | BRCA.LumA |
| TCGA-AN-A0XO | BRCA | C1 | BRCA.LumA |
| TCGA-AO-A03O | BRCA | C2 | BRCA.LumB |
| TCGA-AO-A03T | BRCA | C2 | BRCA.Normal |
| TCGA-AO-A03U | BRCA | C3 | BRCA.Normal |
| TCGA-AO-A0J3 | BRCA | C4 | BRCA.LumB |
| TCGA-AO-A12C | BRCA | C1 | BRCA.LumA |
| TCGA-AO-A1KS | BRCA | C4 | BRCA.LumB |
| TCGA-AQ-A54O | BRCA | C1 | BRCA.LumB |
| TCGA-AR-A0TU | BRCA | C2 | BRCA.Basal |
| TCGA-AR-A1AI | BRCA | C2 | BRCA.Basal |
| TCGA-AR-A24Q | BRCA | C1 | BRCA.Basal |
| TCGA-AR-A24Z | BRCA | C2 | BRCA.LumB |
| TCGA-AR-A250 | BRCA | C1 | BRCA.LumB |
| TCGA-B6-A0I5 | BRCA | C1 | BRCA.LumA |
| TCGA-B6-A0I8 | BRCA | C3 | BRCA.LumA |
| TCGA-B6-A0I9 | BRCA | C1 | BRCA.Her2 |
| TCGA-B6-A0IM | BRCA | C1 | BRCA.LumA |
| TCGA-B6-A0IP | BRCA | C3 | BRCA.LumA |
| TCGA-B6-A0RT | BRCA | C2 | BRCA.Basal |
| TCGA-B6-A2IU | BRCA | C3 | BRCA.LumA |
| TCGA-BH-A0B1 | BRCA | C2 | BRCA.LumA |
| TCGA-BH-A0B2 | BRCA | C2 | BRCA.Normal |
| TCGA-BH-A0BD | BRCA | C2 | BRCA.LumB |
| TCGA-BH-A0DD | BRCA | C2 | BRCA.LumB |
| TCGA-BH-A0DP | BRCA | C3 | BRCA.Normal |
| TCGA-BH-A0E7 | BRCA | C3 | BRCA.LumA |
| TCGA-BH-A0HB | BRCA | C2 | BRCA.LumA |
| TCGA-BH-A0HU | BRCA | C1 | BRCA.LumB |
| TCGA-BH-A0WA | BRCA | C2 | BRCA.Basal |
| TCGA-BH-A18K | BRCA | C1 | BRCA.Normal |
| TCGA-BH-A1FH | BRCA | C1 | BRCA.Normal |
| TCGA-BH-A203 | BRCA | C1 | BRCA.Normal |
| TCGA-BH-A42U | BRCA | C3 | BRCA.Normal |
| TCGA-C8-A12Q | BRCA | C1 | BRCA.Her2 |
| TCGA-C8-A130 | BRCA | C2 | BRCA.LumB |
| TCGA-C8-A131 | BRCA | C1 | BRCA.Basal |
| TCGA-C8-A1HF | BRCA | C2 | BRCA.Her2 |
| TCGA-C8-A1HI | BRCA | C2 | BRCA.LumA |
| TCGA-C8-A26W | BRCA | C2 | BRCA.LumB |
| TCGA-C8-A27B | BRCA | C2 | BRCA.Basal |
| TCGA-D8-A141 | BRCA | C6 | BRCA.LumA |
| TCGA-D8-A1JI | BRCA | C3 | BRCA.LumB |
| TCGA-D8-A1JK | BRCA | C2 | BRCA.Basal |
| TCGA-D8-A1JT | BRCA | C4 | BRCA.LumB |
| TCGA-D8-A1X5 | BRCA | C3 | BRCA.LumB |
| TCGA-D8-A1XB | BRCA | C1 | BRCA.LumA |
| TCGA-D8-A1XC | BRCA | C3 | BRCA.LumA |
| TCGA-D8-A1XW | BRCA | C3 | BRCA.Normal |
| TCGA-D8-A1Y0 | BRCA | C2 | BRCA.LumA |
| TCGA-D8-A27I | BRCA | C3 | BRCA.LumA |
| TCGA-E2-A10F | BRCA | C1 | BRCA.LumA |
| TCGA-E2-A15A | BRCA | C2 | BRCA.LumB |
| TCGA-E2-A15R | BRCA | C3 | BRCA.LumA |
| TCGA-E2-A1IO | BRCA | C3 | BRCA.LumA |
| TCGA-E2-A1L8 | BRCA | C1 | BRCA.LumA |
| TCGA-E2-A3DX | BRCA | C3 | BRCA.LumA |
| TCGA-E2-A572 | BRCA | C4 | BRCA.LumA |
| TCGA-E9-A1N9 | BRCA | C1 | BRCA.Normal |
| TCGA-E9-A1R0 | BRCA | C3 | BRCA.LumA |
| TCGA-E9-A1RC | BRCA | C1 | BRCA.LumA |
| TCGA-E9-A1RI | BRCA | C1 | BRCA.LumA |
| TCGA-E9-A3QA | BRCA | C2 | BRCA.Basal |
| TCGA-EW-A2FS | BRCA | C2 | BRCA.LumA |
| TCGA-EW-A6SA | BRCA | C1 | BRCA.LumB |
| TCGA-GM-A2DI | BRCA | C3 | BRCA.LumA |
| TCGA-GM-A5PX | BRCA | C3 | BRCA.LumA |
| TCGA-LD-A66U | BRCA | C1 | BRCA.LumA |
| TCGA-LL-A740 | BRCA | C1 | BRCA.LumA |
| TCGA-MS-A51U | BRCA | C6 | BRCA.LumA |
| TCGA-OL-A5D8 | BRCA | C2 | BRCA.LumA |
| TCGA-OL-A5S0 | BRCA | C2 | BRCA.Basal |
| TCGA-3C-AALK | BRCA | C1 | BRCA.LumA |
| TCGA-A2-A04R | BRCA | C1 | BRCA.LumB |
| TCGA-A2-A04W | BRCA | C1 | BRCA.Her2 |
| TCGA-A2-A0CW | BRCA | C2 | BRCA.LumB |
| TCGA-A2-A0ES | BRCA | C3 | BRCA.LumA |
| TCGA-A2-A1FW | BRCA | C4 | BRCA.LumB |
| TCGA-A2-A4S1 | BRCA | C6 | BRCA.Basal |
| TCGA-A7-A2KD | BRCA | C2 | BRCA.LumB |
| TCGA-A7-A3IZ | BRCA | C3 | BRCA.LumA |
| TCGA-A7-A4SE | BRCA | C2 | BRCA.Basal |
| TCGA-A8-A06Y | BRCA | C4 | BRCA.LumA |
| TCGA-A8-A07B | BRCA | C1 | BRCA.LumA |
| TCGA-A8-A07I | BRCA | C2 | BRCA.Her2 |
| TCGA-A8-A07O | BRCA | C1 | BRCA.Basal |
| TCGA-A8-A07U | BRCA | C2 | BRCA.Basal |
| TCGA-A8-A083 | BRCA | C4 | BRCA.LumA |
| TCGA-AC-A23G | BRCA | C1 | BRCA.LumA |
| TCGA-AC-A2FK | BRCA | C3 | BRCA.Normal |
| TCGA-AC-A2QH | BRCA | C4 | BRCA.Basal |
| TCGA-AC-A7VB | BRCA | C2 | BRCA.LumB |
| TCGA-AC-A8OS | BRCA | C3 | BRCA.LumA |
| TCGA-AN-A04C | BRCA | C1 | BRCA.Her2 |
| TCGA-AN-A0AK | BRCA | C1 | BRCA.LumB |
| TCGA-AN-A0AM | BRCA | C2 | BRCA.LumB |
| TCGA-AN-A0AR | BRCA | C1 | BRCA.Basal |
| TCGA-AN-A0FK | BRCA | C1 | BRCA.LumA |
| TCGA-AN-A0FW | BRCA | C1 | BRCA.LumA |
| TCGA-AO-A03N | BRCA | C1 | BRCA.LumB |
| TCGA-AO-A0JM | BRCA | C2 | BRCA.LumB |
| TCGA-AO-A125 | BRCA | C3 | BRCA.LumA |
| TCGA-AO-A126 | BRCA | C1 | BRCA.LumA |
| TCGA-AO-A128 | BRCA | C2 | BRCA.Basal |
| TCGA-AO-A1KT | BRCA | C1 | BRCA.LumB |
| TCGA-AR-A1AM | BRCA | C1 | BRCA.LumA |
| TCGA-AR-A24M | BRCA | C6 | BRCA.LumA |
| TCGA-AR-A2LO | BRCA | C3 | BRCA.LumA |
| TCGA-B6-A0I2 | BRCA | C2 | BRCA.Basal |
| TCGA-B6-A0I6 | BRCA | C1 | BRCA.Basal |
| TCGA-B6-A0IJ | BRCA | C2 | BRCA.Basal |
| TCGA-B6-A0RL | BRCA | C4 | BRCA.LumB |
| TCGA-B6-A0RP | BRCA | C3 | BRCA.LumA |
| TCGA-B6-A0RU | BRCA | C1 | BRCA.Basal |
| TCGA-B6-A0WT | BRCA | C1 | BRCA.LumA |
| TCGA-B6-A402 | BRCA | C1 | BRCA.Basal |
| TCGA-B6-A409 | BRCA | C2 | BRCA.Basal |
| TCGA-BH-A0BS | BRCA | C4 | BRCA.Normal |
| TCGA-BH-A1EV | BRCA | C1 | BRCA.Normal |
| TCGA-BH-A1EW | BRCA | C2 | BRCA.LumA |
| TCGA-BH-A1F2 | BRCA | C4 | BRCA.Normal |
| TCGA-BH-A1FC | BRCA | C2 | BRCA.Normal |
| TCGA-BH-A1FE | BRCA | C2 | BRCA.Normal |
| TCGA-BH-A2L8 | BRCA | C2 | BRCA.LumA |
| TCGA-C8-A12T | BRCA | C1 | BRCA.Her2 |
| TCGA-C8-A12W | BRCA | C1 | BRCA.LumB |
| TCGA-C8-A132 | BRCA | C1 | BRCA.LumA |
| TCGA-C8-A3M8 | BRCA | C1 | BRCA.LumB |
| TCGA-D8-A1J9 | BRCA | C4 | BRCA.LumB |
| TCGA-D8-A1JC | BRCA | C2 | BRCA.LumB |
| TCGA-D8-A1JD | BRCA | C1 | BRCA.LumB |
| TCGA-D8-A1JJ | BRCA | C1 | BRCA.LumB |
| TCGA-D8-A1JL | BRCA | C2 | BRCA.Basal |
| TCGA-D8-A1JM | BRCA | C1 | BRCA.Basal |
| TCGA-D8-A1XK | BRCA | C2 | BRCA.Basal |
| TCGA-D8-A1XZ | BRCA | C2 | BRCA.LumB |
| TCGA-D8-A3Z5 | BRCA | C3 | BRCA.LumA |
| TCGA-E2-A14Q | BRCA | C1 | BRCA.LumA |
| TCGA-E2-A14T | BRCA | C3 | BRCA.LumA |
| TCGA-E2-A15F | BRCA | C2 | BRCA.LumA |
| TCGA-E2-A15H | BRCA | C1 | BRCA.LumA |
| TCGA-E2-A15I | BRCA | C3 | BRCA.Normal |
| TCGA-E2-A1LH | BRCA | C2 | BRCA.Normal |
| TCGA-E2-A1LI | BRCA | C2 | BRCA.Basal |
| TCGA-E9-A1NE | BRCA | C2 | BRCA.LumA |
| TCGA-E9-A1NH | BRCA | C3 | BRCA.LumA |
| TCGA-E9-A1R3 | BRCA | C6 | BRCA.LumA |
| TCGA-E9-A1RG | BRCA | C1 | BRCA.LumB |
| TCGA-E9-A5UP | BRCA | C4 | BRCA.LumA |
| TCGA-EW-A1J5 | BRCA | C2 | BRCA.LumA |
| TCGA-EW-A1OV | BRCA | C2 | BRCA.Her2 |
| TCGA-EW-A1P0 | BRCA | C4 | BRCA.LumB |
| TCGA-EW-A1P8 | BRCA | C2 | BRCA.Basal |
| TCGA-EW-A1PA | BRCA | C1 | BRCA.LumA |
| TCGA-EW-A1PE | BRCA | C2 | BRCA.LumA |
| TCGA-EW-A1PG | BRCA | C1 | BRCA.Normal |
| TCGA-EW-A423 | BRCA | C3 | BRCA.LumA |
| TCGA-EW-A424 | BRCA | C1 | BRCA.LumA |
| TCGA-GM-A2D9 | BRCA | C1 | BRCA.LumA |
| TCGA-GM-A2DC | BRCA | C3 | BRCA.LumA |
| TCGA-GM-A2DN | BRCA | C2 | BRCA.LumA |
| TCGA-HN-A2OB | BRCA | C3 | BRCA.LumA |
| TCGA-LD-A7W6 | BRCA | C2 | BRCA.LumA |
| TCGA-LD-A9QF | BRCA | C2 | BRCA.Normal |
| TCGA-LL-A50Y | BRCA | C1 | BRCA.LumA |
| TCGA-LL-A5YL | BRCA | C1 | BRCA.LumA |
| TCGA-LL-A6FQ | BRCA | C2 | BRCA.LumA |
| TCGA-LL-A73Z | BRCA | C2 | BRCA.Normal |
| TCGA-LL-A9Q3 | BRCA | C1 | BRCA.LumA |
| TCGA-OL-A5RZ | BRCA | C1 | BRCA.Her2 |
| TCGA-OL-A66I | BRCA | C2 | BRCA.Basal |
| TCGA-OL-A6VO | BRCA | C2 | BRCA.Basal |
| TCGA-PE-A5DD | BRCA | C1 | BRCA.LumA |
| TCGA-PL-A8LV | BRCA | C1 | BRCA.Basal |
| TCGA-S3-AA14 | BRCA | C1 | BRCA.LumA |
| TCGA-WT-AB41 | BRCA | C2 | BRCA.LumA |
| TCGA-Z7-A8R5 | BRCA | C1 | BRCA.LumA |

|  |  |  |  |
| --- | --- | --- | --- |
